# Human hippocampal and entorhinal neurons encode the temporal structure of experience

**DOI:** 10.1038/s41586-024-07973-1

**Published:** 2024-09-25

**Authors:** Pawel Tacikowski, Güldamla Kalender, Davide Ciliberti, Itzhak Fried

**Affiliations:** 1 Department of Neurosurgery, University of California, Los Angeles, Los Angeles, CA USA; 2 Department of Neuroscience, Karolinska Institutet, Stockholm, Sweden; 3 Coimbra Institute for Biomedical Imaging and Translational Research, University of Coimbra, Coimbra, Portugal; 4 Department of Psychiatry and Biobehavioral Sciences, University of California, Los Angeles, Los Angeles, CA USA; 5 Faculty of Medicine, Tel Aviv University, Tel Aviv, Israel; 6 Present Address: Department of Psychiatry and Behavioral Sciences, University of Minnesota, Minneapolis, MN USA; 7 Present Address: Institute of Cognitive Neurology and Dementia Research, Otto-von-Guericke University Magdeburg, Magdeburg, Germany

**Keywords:** Learning and memory, Cognitive neuroscience

## Abstract

Extracting the underlying temporal structure of experience is a fundamental aspect of learning and memory that allows us to predict what is likely to happen next. Current knowledge about the neural underpinnings of this cognitive process in humans stems from functional neuroimaging research^1–5^. As these methods lack direct access to the neuronal level, it remains unknown how this process is computed by neurons in the human brain. Here we record from single neurons in individuals who have been implanted with intracranial electrodes for clinical reasons, and show that human hippocampal and entorhinal neurons gradually modify their activity to encode the temporal structure of a complex image presentation sequence. This representation was formed rapidly, without providing specific instructions to the participants, and persisted when the prescribed experience was no longer present. Furthermore, the structure recovered from the population activity of hippocampal–entorhinal neurons closely resembled the structural graph defining the sequence, but at the same time, also reflected the probability of upcoming stimuli. Finally, learning of the sequence graph was related to spontaneous, time-compressed replay of individual neurons’ activity corresponding to previously experienced graph trajectories. These findings demonstrate that neurons in the hippocampus and entorhinal cortex integrate the ‘what’ and ‘when’ information to extract durable and predictive representations of the temporal structure of human experience.

## Abstract (web summary)

Single-neuron recordings from intracranial electrodes inserted into human brains for clinical reasons suggest that the temporal structure of human experience is encoded in human hippocampal and entorhinal neurons.

## Main

Extracting temporal patterns of recurring events is fundamentally important for organizing information in memory, predicting the future and guiding flexible behaviours^6,7^. How this process is carried out by neurons in the human brain remains unknown. Studies on spatial navigation provide some important clues, as moving through space essentially corresponds to a sequence of visiting locations characterized by specific neuronal signatures. A ‘cognitive map’ of the spatial environment is encoded by a range of interacting neuron types, including hippocampal ‘place cells’ that fire when the animal is at a specific location^8,9^ and entorhinal ‘grid cells’ that provide a metric of spatial distance^10,11^. Remarkably, the brain uses similar neural principles to represent non-spatial features, such as sound frequency^12^, object characteristics^13^, abstract space^14^ and time^15,16^. This cognitive map is predictive, in that it informs about future states that the agent is likely to experience^17–20^. The fact that hippocampal–entorhinal neurons represent relations between features of information and encode time makes this brain circuit an ideal candidate system to extract the temporal structure of experience. Functional neuroimaging research in humans generally supports this view^1–5^, but how such extraction is achieved by hippocampal–entorhinal neurons remains unknown.

**Fig. 1:**
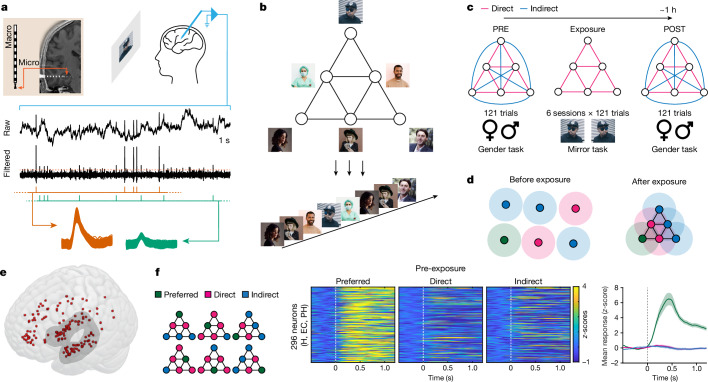
Experimental procedures and selective neurons. **a**, Top, extracellular spiking activity was recorded from eight microwires extending from the tip of each depth macro-electrode. There were 7–12 macro-electrodes per patient. Raw local field potential signal was high-pass filtered and thresholded to detect spiking activity. Bottom, spike waveforms from one whole recording session, grouped into two clusters (two putative neurons) based on the waveforms’ amplitude and shape. **b**, The sequence of stimuli presentation (bottom) corresponded to a ‘random walk’ on a pyramid graph (top) so that only images directly linked on the graph were displayed immediately after another. **c**, The participant’s task was either to determine whether each displayed image shows a male or a female (gender task; PRE and POST) or whether the image is the same or mirrored when compared to PRE (E1–E6). **d**, A schematic representation of the hypothesis. Circles represent ‘place fields’ of selective neurons in abstract space. Before exposure, each neuron responds preferentially to a different image, and the arrangement of place fields is largely random. After exposure to the pyramid structure, the green neuron should respond more strongly to images directly linked on the pyramid to its preferred stimulus (magenta) than to images linked indirectly (blue). The same logic applies to all nodes, regardless of whether the ‘seed’ is at an inner or an outer node (see **f**). **e**, Neuronal activity was recorded from multiple brain regions, including the hippocampal–entorhinal system and amygdalae (shaded area). Dots represent localizations of microwires where putative neurons were detected. These localizations are overlaid on the 152-MNI-T1 3D template brain rendered by MRIcroGL software. **f**, A significant proportion of selective neurons was found in the hippocampus (H), entorhinal cortex (EC) and parahippocampal gyrus (PH). Each row of the heat maps shows the mean spiking activity of one neuron during PRE (*z*-scored and baseline-corrected; −0.5 to 0 s). The plot on the right shows mean responses ± s.e.m. from all selective neurons. Note that owing to copyright issues, all original images used in the study were replaced in this and all subsequent figures by comparable free stock photos. The original images are available from the corresponding authors.

Here we recorded extracellular spiking activity from 17 patients with epilepsy who were implanted with intracranial depth electrodes for clinical reasons^21^ (Fig. 1a and Supplementary Table 1; 21 recording sessions). Our experimental paradigm capitalized on the fact that the human medial temporal lobe (MTL) contains neurons that respond selectively to particular people^22,23^. For each participant, we selected six images that were associated with preferential neuronal responses in the preceding screening experiment. Each image was then arbitrarily assigned to a different location on a pyramid graph (Fig. 1b). There were three main study phases: pre-exposure (PRE), exposure, and post-exposure (POST) (Fig. 1c). During PRE (baseline), images were displayed in pseudo random order (60 direct and 60 indirect graph-transitions). During the subsequent six exposure phases (E1–E6), the order of image presentations was determined by the pyramid graph, so that only images directly linked on the graph were displayed immediately one after another (Fig. 1c). Finally, POST (read-out) was identical to PRE; during this phase, there was no pyramid rule in the sequence of image presentations. During every phase, the participants performed behavioural tasks that were unrelated to the temporal pyramid rule (Fig. 1c). We hypothesized that hippocampal–entorhinal neurons gradually represent the temporal pyramid structure by responding in an increasingly similar manner to stimuli directly linked on the graph. Note that the configuration of directly and indirectly connected nodes is different, depending on whether the seed is an inner or an outer node (Fig. 1d,f).

## Individual neurons

**Extended Data Fig. 1:**
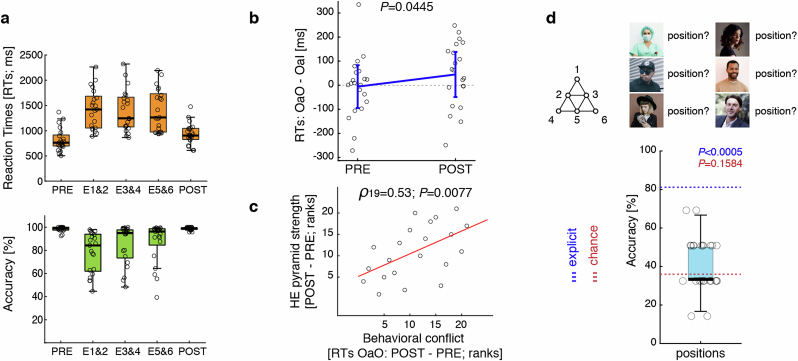
Behavioural performance. (**a**) The participants responded more slowly and committed more errors in the ‘mirror task’ than in the ‘gender task’ (combined data from E1-E6 versus combined data from PRE and POST; *P* = 5.96 × 10^−5^ and *P* = 5.95 × 10^−5^, respectively; *n* = 21 sessions; Wilcoxon signed-rank tests; two-sided). (**b**) During exposure, the outer nodes could never immediately follow one another. If the participants extracted this rule, outer-after-outer trials (OaO) during POST should be unexpected and thus related to longer reaction times (RTs) than outer-after-inner trials (OaI). Indeed, we found that the difference between RTs in OaO minus OaI trials increased in POST compared to PRE (*P* = 0.0445; 10,000 permutations of PRE and POST labels). The plot shows means ± s.e.m. Circles correspond to datapoints from individual sessions (*n* = 21). The *P*-value was calculated as a number of permutations with a higher difference than the one actually detected, divided by the total number of permutations. (**c**) The above-mentioned behavioural conflict was especially pronounced among the patients who developed a robust hippocampal-entorhinal representation of the pyramid, calculated per participant as similarity between neuronal population responses and the successor template in POST (see Fig. 3g; ρ_19_ = 0.53; *P* = 0.0077; Spearman correlation with 10,000 permutations of the session order). Another index of behavioural conflict—RTs in trials after ‘indirect’ transitions—was also positively correlated with the strength of the hippocampal-entorhinal successor representation (indirect trials: POST minus PRE; ρ_19_ = 0.39; *P* = 0.04; Spearman correlation with 10,000 permutations of the session order). In contrast, RTs in trials without conflict (i.e., ‘direct’ and OaI transitions; POST minus PRE) did not significantly correlate with the strength of the hippocampal-entorhinal successor representation (ρ_19_ = 0.32; *P* = 0.0814 and ρ_19_ = 0.21; *P* = 0.173, respectively). All RT analyses were performed on correct trials only. Very short (<200 ms) and very long (> 5000 ms) RTs were discarded. The above *P* values were calculated as a number of permutations with a higher correlation coefficient than the one actually detected, divided by the total number of permutations. (**d**) Twenty-five healthy controls (see Methods) completed the same behavioural procedure as the patients. They were then asked an open question: ‘Have you noticed any pattern in the sequence of images?’ None of the participants reported noticing a graph-like organization of the sequence. Then, we informed them about the underlying structure and asked them to assign each image to a specific node (the ‘positions’ task). The pyramid has six variants (three rotations and two flips). The ‘positions accuracy’ was calculated as the maximum number of hits. For example, if someone’s highest score was three out of six for one variant and less than three hits for other variants, this person’s accuracy score was 50%. To calculate the ‘links accuracy,’ we checked whether each pair of images was linked directly or indirectly on the graph provided by each participant and compared it to the actual pyramid. Similarly, we calculated Spearman correlation coefficients (Fisher-transformed) between pairwise distances provided by each participant and the actual pairwise distances (‘distance similarity’ index). Finally, we checked how often the participants assigned the correct images to the inner versus outer nodes (‘inner-outer accuracy’). For each index, the chance level was estimated as the mean performance of 10,000 randomly generated ‘participants’ (red dashed line). To establish the ‘explicit benchmark’ (blue dashed line), we tested another eight control participants (see Methods). From the beginning, we informed them that the sequence of images during exposure phases will follow the pyramid graph, but we did not explain which image is located where on the graph. All other aspects of the procedure and analysis were the same as explained above. The mean performance of this additional group served as the explicit benchmark. We found that ‘positions accuracy’ did not significantly differ from chance level and was significantly below the explicit benchmark (*P* = 0.1584 and *P* = 8.54 × 10^−6^, respectively). Other indexes, arguably referring to less detailed knowledge of the graph, were significantly above chance level, but still below the explicit benchmark (‘links accuracy’ and ‘distance similarity’ versus chance: *P* = 0.007; ‘links accuracy’ and ‘distance similarity’ versus explicit: *P* = 7.04 × 10^−6^; ‘inner-outer accuracy’ versus chance: *P* = 0.0186; ‘inner-outer accuracy’ versus explicit: *P* = 0.0004). All the above *P* values are from the Wilcoxon signed-rank tests (two-sided). Together, these results suggest that the healthy control participants (and patients) did not have detailed explicit knowledge of the pyramid. The central marks of the box plots (panels a and d) indicate the medians. The bottom and top edges of the boxes indicate the 25th (Q1) and 75th (Q3) percentiles, respectively. The whiskers extend to the most extreme data points not considered outliers (1.5 × interquartile range above Q3 or below Q1).

**Extended Data Fig. 2:**
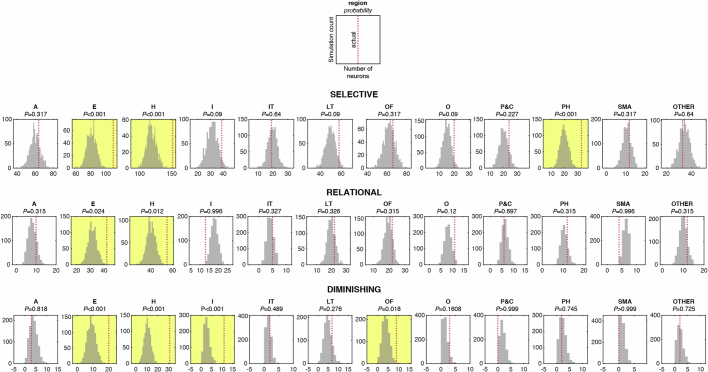
Proportion of selective, relational, and diminishing selectivity neurons across the brain regions. The number of ‘selective neurons’ was significantly above chance level in the hippocampus (H), entorhinal cortex (E), and parahippocampal gyrus (PH). The number of ‘relational neurons’ was significantly above chance only in the hippocampus (H) and entorhinal cortex (E). The number of ‘diminishing selectivity neurons’ was significantly above chance level in the hippocampus (H), entorhinal cortex (E), insula (I), and orbitofrontal cortex (OF). Histograms show the number of units of a given type obtained from 1,000 random permutations of stimuli or phase labels (see Methods). *P* values were calculated as the number of permutations with more neurons of a given type than the number of neurons actually detected, divided by the total number of permutations. If in none of the permutations the number of neurons was above the actual count, the *P* < 0.001 range is reported. No adjustment for multiple comparisons was applied. Dashed lines represent the actual number of neurons detected. ***Abbreviations:*** A – amygdala; E – entorhinal cortex; H – hippocampus; I – insula and operculum; IT – inferior temporal cortex; LT – lateral temporal cortex; OF – orbitofrontal cortex and anterior cingulate cortex; O – occipital cortex; P&C – parietal cortex & middle/posterior cingulate cortex; PH – parahippocampal gyrus; SMA – Supplementary Motor Area.

Altogether, we identified 1,456 single- and multi-units (hereafter called ‘neurons’) across multiple brain regions (Fig. 1e and Supplementary Table 2). The unit yield was generally high and comparable across the participants (minimum = 27, maximum = 118, average = 69; Supplementary Table 2). We first identified selective neurons that responded significantly more strongly to one stimulus than to all other stimuli during PRE (Methods). Note that selectivity was defined in a narrow sense, only relative to other images used in the current study. We found a significant proportion of selective neurons in the hippocampus, entorhinal cortex and parahippocampal gyrus (Fig. 1f, Extended Data Fig. 2 and Supplementary Tables 3 and 4; *n* = 152, *n* = 111, and *n* = 33, respectively; 45%, 53%, and 56% of all identified neurons from those regions, respectively; *P* < 0.001 above chance level for all three regions). Depending on the position of the preferred stimulus on the pyramid graph, we classified the remaining stimuli as ‘direct’ or ‘indirect’ for a given neuron and used these labels consistently to analyse the subsequent study phases. On average, each node on the graph was associated with preferential responses of 49 selective neurons (minimum = 33, maximum = 64; across all recording sessions). Behavioural data showed that the participants generally followed the instructions and completed the experimental tasks successfully (Extended Data Fig. 1a). Furthermore, stimuli transitions during POST that violated the sequence rules from exposure phases were related to increased response latencies, which suggests that the patients extracted the pyramid graph and used it to guide their behaviour, despite the lack of specific instructions to do so and the task-irrelevant nature of the pyramid (Extended Data Fig. 1b). At the same time, when asked, “Have you noticed any pattern in the stimulus sequence?” none of the patients reported noticing a graph-like organization of the states. A separate behavioural study conducted on twenty-five healthy controls further supported the lack of detailed explicit knowledge of the pyramid structure by the participants after completing the same version of the task as the patients (Extended Data Fig. 1d). Together, the above results validate our methodological approach and show that learning the pyramid was largely implicit.

**Fig. 2:**
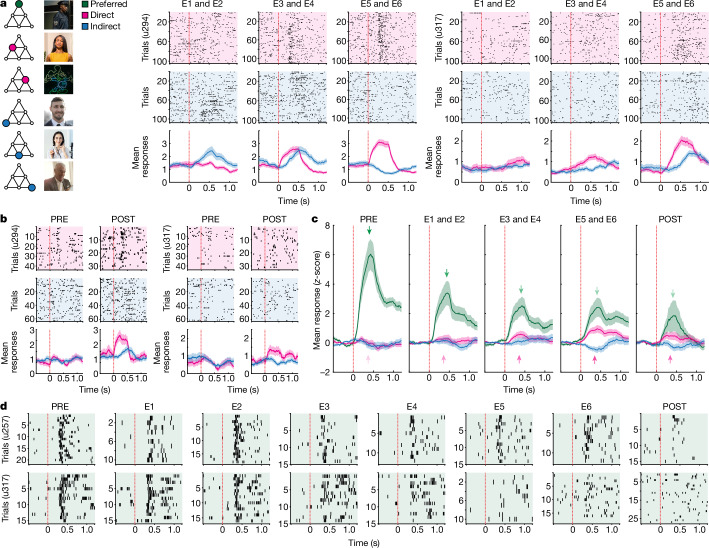
Relational neurons in the hippocampal–entorhinal formation. **a**, Two representative hippocampal neurons that responded preferentially to the image of the policeman (left) during PRE. With exposure to the pyramid rule, they began to respond more strongly to images directly linked to their preferred stimulus on the graph (direct) than to images linked indirectly (indirect). **b**, These two neurons continued to show the same pattern of responses during POST, when the pyramid rule had stopped and the behavioural task had changed. **c**, Average responses (±s.e.m.) of all relational neurons in the hippocampal–entorhinal region (*n* = 97). Apart from showing increasingly stronger responses to direct images, these neurons showed gradually diminishing selectivity for their preferred stimulus. Each neuron’s responses were *z*-scored and baseline-corrected (−0.5 to 0 s). **d**, Two representative neurons showing diminishing selectivity (the bottom panel shows the same neuron as **a**, right). Raster plots in **a**,**b**,**d** show individual spikes during each stimulus presentation. Line plots in **a**,**b** show the mean number of spikes ± s.e.m. Neurons’ identifiers are provided in round brackets.

**Extended Data Fig. 3:**
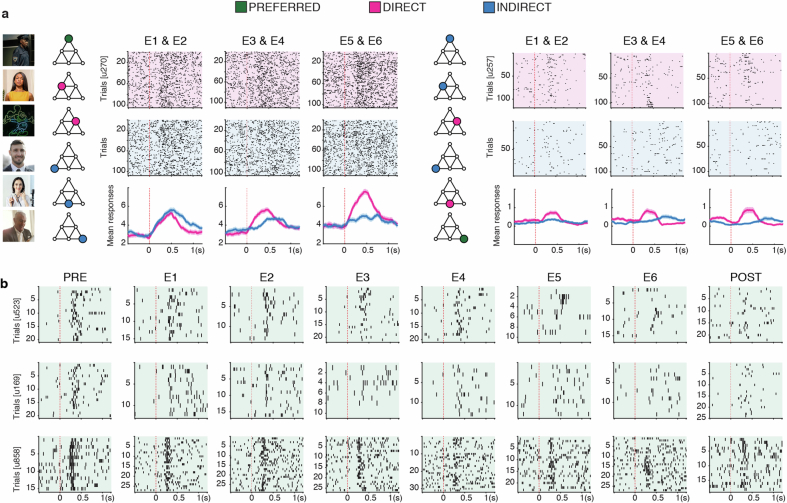
Other examples of relational and diminishing selectivity neurons. (**a**) Throughout the study, these hippocampal relational neurons showed increasingly more robust responses to stimuli that were directly linked to their preferred image on the pyramid graph (magenta) than to images linked indirectly (blue). (**b**) During the study, these diminishing selectivity neurons decreased responses to their preferred stimulus. Raster plots show individual spikes in each trial. Line plots show the mean number of spikes ± s.e.m. Top and middle panels show hippocampal neurons. The bottom panel shows a neuron from the entorhinal cortex.

**Extended Data Fig. 4:**
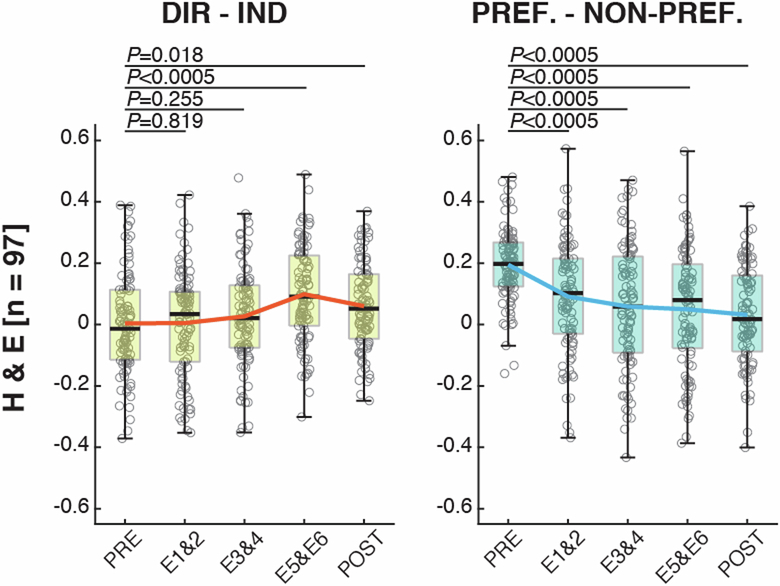
Average responses of all hippocampal-entorhinal relational neurons. These neurons showed significantly stronger responses to direct than indirect images in the late-exposure phases (E5&6) and in POST (left panel; Wilcoxon signed-rank tests against PRE; two-sided; FDR-corrected). These neurons also gradually diminished their selectivity to the preferred image (right panel; Wilcoxon signed-rank tests against PRE; two-sided; FDR-corrected). It is highly unlikely that this diminished selectivity was due to ‘regression toward the mean,’ as responses to preferred stimuli continued to decrease in the subsequent phases (E3&4, E5&6, POST versus E1&2; *P* = 0.0346, *P* = 0.0315, and *P* = 0.0271, respectively; Wilcoxon signed-rank tests; one-sided; FDR-corrected). Each circle corresponds to one neuron. For the definition of box plots, please see the legend of the Extended Data Fig. 1. Solid lines represent means.

Moving on to the main analysis, we identified temporal ‘relational neurons’ that increased their responses to direct stimuli throughout the study (Methods). We found a significant proportion of such neurons specifically in the entorhinal cortex and hippocampus (Extended Data Fig. 2 and Supplementary Tables 3 and 5; *n* = 42 and *n* = 55, respectively; 20% and 16% of all identified neurons in those regions, respectively; *P* = 0.024 and *P* = 0.012 above the chance level, respectively). Figure 2a shows two representative relational neurons from the right hippocampus (see also Extended Data Fig. 3a). Of note, these two cells continued to respond more strongly to direct stimuli even during POST, when the order of image presentations no longer followed the pyramid rule and when the behavioural task had changed (Fig. 2b). Responses of all hippocampal–entorhinal relational neurons to direct stimuli were significantly stronger during late experiment phases (E5 and E6) than during PRE, and significantly stronger during POST than during PRE (Fig. 2c and Extended Data Fig. 4; *P* = 3.56 × 10^−5^ and *P* = 0.018, respectively; two-sided Wilcoxon signed-rank test, false discovery rate (FDR)-corrected; note that data from PRE and POST were not used in the statistical selection of these neurons—thus, the above results are not biased by the selection criterion;  Methods). Notably, relational neurons also gradually decreased their responses to preferred stimuli (Fig. 2c,d and Extended Data Fig. 4; comparisons versus PRE; E1 and E2: *P* = 1.98 × 10^−6^; E3 and E4: *P* = 2.26 × 10^−7^; E5 and E6: *P* = 9.69 × 10^−9^; POST: *P* = 1.07 × 10^−9^; two-sided Wilcoxon signed-rank tests, FDR-corrected; for general results on neurons that gradually decreased their selectivity, see Extended Data Figs. 2 and 3b and Supplementary Tables 3 and 6). The above results support our hypothesis by demonstrating that hippocampal–entorhinal neurons that initially responded preferentially to one image gradually embedded the pyramid graph, by showing diminished selectivity to that image and increased responses to adjacent stimuli.

## Population code

**Fig. 3:**
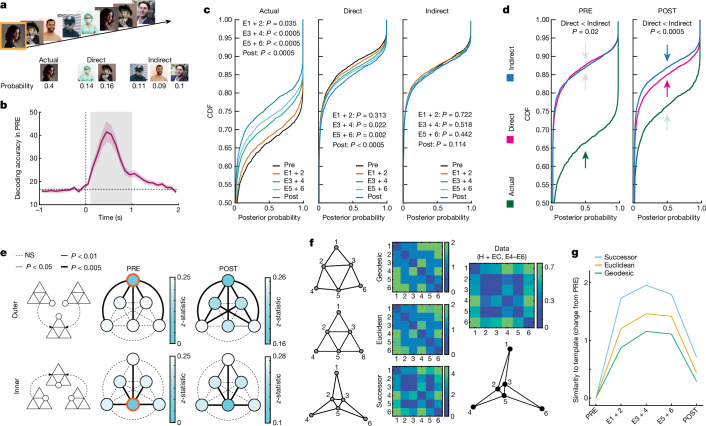
Hippocampal–entorhinal population activity remaps to structured experience. **a**, The logic behind the population decoding analysis. **b**, Neuronal responses contained enough information to successfully decode the stimulus identity during PRE (chance level ≈ 17%; data from all identified neurons; *n* = 1,456). The plot shows mean decoding accuracy (±s.e.m.) from 100-ms bins averaged across all recording sessions (*n* = 21; time zero is the stimulus onset). The shaded grey area marks the time window used for further analyses. **c**, Results from the hippocampal–entorhinal neurons (*n* = 546). *P* values obtained from the Kolmogorov–Smirnov tests between cumulative distribution functions (CDFs) of posterior probabilities assigned by the decoder during PRE versus subsequent study phases (one-sided). **d**, The difference between CDFs for direct and indirect stimuli remained significant during POST (Kolmogorov–Smirnov test; one-sided). **e**, Top row, combined data from trials where the actually presented stimulus was at an outer node of the pyramid. *P* values represented by dotted or solid lines of different widths were obtained from Kolmogorov–Smirnov tests between each pair of nodes (one-sided; FDR-corrected). Colour intensities correspond to distances (Kolmogorov–Smirnov *z*-statistic) between the respective CDFs. The seed node is marked in orange. Bottom row, analogous results for trials where the stimulus actually presented was at an inner node. NS, not significant. **f**, Distance matrixes and graphs corresponding to the geodesic, Euclidean and successor templates. Each graph shows the most faithful 2D representation of the respective distance matrix using the multidimensional scaling analysis. Note that the matrix and graph obtained from the neuronal data (right) closely resemble the successor template (546 hippocampal–entorhinal neurons; E4–E6 data combined for illustration purposes). **g**, The degree of similarity between data and each template throughout the study. Spearman’s correlation coefficients (Fisher-transformed) between each template and neural data from respective phases (changes from PRE).

**Extended Data Fig. 5:**
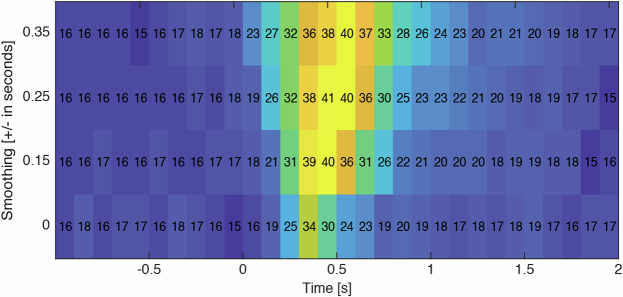
Population activity during PRE contained enough information to decode stimulus identity above the chance level. We used the Poisson Naive Bayes classifier to decode stimulus identity during PRE (‘leave-one-out’ cross-validation). This analysis was performed on data from all 1456 neurons. The highest decoding accuracy was obtained for the moving sum of ± 0.25 s and the 0.1 – 1 s time window. These parameters were used in the remaining analyses.

**Extended Data Fig. 6:**
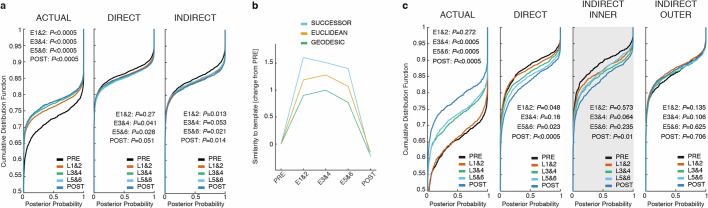
Additional neuronal population results. (**a**) Population decoding results from all neurons outside of the hippocampal-entorhinal system (*n* = 910; 21 sessions). Plots show CDFs of posterior probabilities assigned to the actual, direct, and indirect images in the subsequent study phases. *P* values obtained from the Kolmogorov-Smirnov tests between CDFs of subsequent study phases versus PRE (one-sided). Exact *P* values for ‘actual’ E1&2: 2.68 × 10^−11^; E3&4: 1.16 × 10^−20^; E5&6: 1.28 × 10^−21^; POST: 3.59 × 10^−18^. (**b**) Reconstruction of the pyramid structure from the activity of all non-hippocampal-entorhinal neurons (*n* = 910; 21 sessions). During exposure, but not during POST, all templates fit the data significantly above the chance level (geodesic: E1&2: *P* = 0.0286; E3&4: *P* = 0.0286; E5&6: *P* = 0.0399; POST: *P* = 0.7053; Euclidean: E1&2: *P* = 0.005; E3&4: *P* = 0.005; E5&6: *P* = 0.0064; POST: *P* = 0.6505; successor: E1&2: *P* < 0.0001; E3&4: *P* = 0.0003; E5&6: *P* = 0.0003; POST: *P* = 0.7169; change from PRE; 10,000 permutations; FDR-corrected). The successor template significantly outperformed the geodesic template during exposure phases but not during POST (successor versus geodesic: E1&2: *P* = 0.0008; E3&4: *P* = 0.0128; E5&6: *P* = 0.0022; POST: *P* = 0.5216; change from PRE; 10,000 permutations; FDR-corrected). The difference between successor and Euclidean templates was non-significant or marginally significant (successor versus Euclidean: E1&2: *P* = 0.0612; E3&4: *P* = 0.1653; E5&6: *P* = 0.0878; POST: *P* = 0.6476; change from PRE; 10,000 permutations; FDR-corrected). The plot shows Spearman’s correlation coefficients (Fisher-transformed) between each template and the neural data from respective phases. *P* values were calculated as the number of permutations with a higher correlation coefficient than the one actually detected, divided by the total number of permutations. If in none of the permutations the correlation was above the actual coefficient, the *P* < 0.0001 range is reported. (**c**) Population decoding results for trials in which the actually presented images were from an outer node of the pyramid (all 546 hippocampal-entorhinal neurons). Plots show CDFs of posterior probabilities assigned to the actual, direct, indirect-inner, and indirect-outer nodes during the subsequent study phases. *P* values obtained from Kolmogorov-Smirnov tests between CDFs in the subsequent study phases versus PRE (one-sided). Exact *P* values for ‘actual’ E1&2: 0.2718; E3&4: 3.59 × 10^−7^; E5&6: 1.98 × 10^−6^; POST: 4.56 × 10^−13^. Exact *P*-value for ‘direct’ POST: 1.53 × 10^−5^.

Next, we tested whether the pyramid representation was robust enough to shift the activity pattern of the entire hippocampal–entorhinal neuronal population. To this end, we used the Bayesian naive classifier to decode stimulus identity during each image presentation (Methods). Instead of simply checking whether decoding was correct, we analysed posterior probabilities that the decoder assigned to the image actually presented (actual), images directly linked to that stimulus on the graph (direct) and images linked indirectly (indirect) (Fig. 3a and Methods). This analysis was performed for each recording session separately because of the different stimuli used, but the resulting probability distributions were combined across all sessions. The classifier was trained on data from PRE and tested on all subsequent study phases (for testing in PRE, we used the ‘leave-one-out’ cross-validation). The analysis was performed on all identified hippocampal and entorhinal neurons, regardless of their selectivity (*n* = 546). We found that the data from PRE contained enough information to decode stimulus identity significantly above the chance level; this important prerequisite makes the analysis of subsequent phases meaningful (Fig. 3b and Extended Data Fig. 5). Over the course of the study, the classifier assigned progressively lower probabilities to the images actually presented (Fig. 3c; comparisons versus PRE; E1 and E2: *P* = 0.035; E3 and E4: *P* = 1.14 × 10^−8^; E5 and E6: *P* = 1.49 × 10^−11^; POST: *P* = 2.02 × 10^−16^). By contrast, the classifier assigned increasingly higher probabilities to stimuli that were directly linked to the actual stimuli on the pyramid graph (Fig. 3c; comparisons versus PRE; E1 and E2: *P* = 0.313; E3 and E4: *P* = 0.022; E5 and E6: *P* = 0.002; POST: *P* = 1.74 × 10^−4^). Probability distributions for indirectly linked stimuli did not change significantly over the course of the study (Fig. 3c; comparisons versus PRE; E1 and E2: *P* = 0.722; E3 and E4: *P* = 0.518; E5 and E6: *P* = 0.442; POST: *P* = 0.114). The difference between distributions for direct and indirect stimuli was significant even during POST, when the order of image presentations did not follow the pyramid structure and the behavioural task had changed (Fig. 3d; POST-direct versus POST-indirect: *P* = 8.61 × 10^−5^). For all the above comparisons, we used Kolmogorov–Smirnov tests (one-sided). It is noteworthy that an analogous control analysis performed on all neurons outside of the hippocampal–entorhinal system did not reveal any consistent evidence of the pyramid representation (Extended Data Fig. 6a; *n* = 910). The above findings validate and go beyond the results from individual neurons, by showing that the pyramid graph representation affected the activity of the entire neuronal population in the hippocampal–entorhinal complex.

Next, we tested whether the neuronal representation of the pyramid graph followed geodesic geometry—that is, whether distances between neuronal responses to different nodes were equivalent to the minimum number of edges connecting these nodes (that is, the ‘shortest path’ distance). If that were the case, there should be: (1) no prominent differences when all direct nodes are compared to each other; and (2) no prominent differences when all indirect nodes are contrasted with each other. Because the pyramid graph is symmetrical, we grouped together all trials where the actually displayed image (seed) was located at one of the three outer nodes of the pyramid and calculated pairwise distances between posterior probability distributions (see previous paragraph) assigned to the seed versus the remaining nodes. This analysis was performed on all hippocampal–entorhinal neurons (*n* = 546), first for each recording session separately and then combined across all sessions. As expected, during PRE, the seed node differed significantly from all other nodes and the other nodes did not differ significantly between each other or differed only marginally (Fig. 3e). During POST, the seed still differed significantly from the remaining nodes, but now, both direct nodes also significantly differed from the indirect-outer nodes (Fig. 3e). Notably, there was no significant difference between either of the direct nodes and the indirect-inner node. Thus, not all indirect nodes changed their representations to a similar degree, suggesting that the neural encoding of the pyramid was not strictly geodesic (see earlier). An analogous analysis for all inner seed nodes combined revealed generally similar results (Fig. 3e; bottom). In line with this, a single-neuron analysis showed that there was a small but significant proportion of hippocampal relational neurons that during the late study phases responded significantly more strongly to indirect-inner than to indirect-outer nodes (*n* = 12; 4% of all hippocampal neurons; *P* = 0.024). A population decoding analysis analogous to Fig. 3c further supported that the decoding probability of indirect-inner nodes changed throughout the study in a similar manner as the decoding probability of direct nodes (Extended Data Fig. 6c). Together, these findings indicate that the population of hippocampal–entorhinal neurons accurately encoded the general layout of the pyramid graph, but this mapping was not strictly geodesic.

## Recovering the entire graph

Next, we tested whether it was possible to reconstruct the entire pyramid structure from the population activity of hippocampal and entorhinal neurons and if so, what geometry that representation followed. To this end, we calculated Euclidean distances between neurons’ responses to each image versus all other images (this was done for all subsequent study phases), and then, compared the resulting distance matrixes to three templates (Fig. 3f). In the ‘geodesic template’, distances between each pair of nodes corresponded to the shortest path (see ‘Population code’). In the ‘Euclidean template’, distances between relevant nodes (1–5, 2–6 and 4–3) were calculated from the Pythagorean theorem. The ‘successor template’ assumed that the pyramid representation is predictive. This idea has been previously formalized as the ‘successor representation’, which informs how often an agent will experience a particular destination state after starting in the initial state^17–20^. In the present study, temporal predictions can be based on the structure of the pyramid itself. Specifically, the length of all possible paths between the different inner nodes is generally shorter than the length of all paths connecting the outer nodes. Thus, during a random walk, the inner nodes are likely to occur closer in time. If the neural representation is predictive, the above regularities should significantly distort the graph’s representation by shortening distances between the inner nodes (Fig. 3f and  Methods).

We found that over the course of the study, all templates improved their fit to the neural data (Fig. 3g; geodesic: E1 and E2: *P* = 0.0231; E3 and E4: *P* = 0.009; E5 and E6: *P* = 0.009; POST: *P* = 0.2335; Euclidean: E1 and E2: *P* = 0.0035; E3 and E4: *P* = 0.0008; E5 and E6: *P* = 0.0008; POST: *P* = 0.1397; successor: E1 and E2: *P* = 0.0001; E3 and E4: *P* < 0.0001; E5 and E6: *P* < 0.0001; POST: *P* = 0.0434; differences from PRE; 10,000 permutations; FDR-corrected;  Methods). However, the successor template significantly outperformed the other templates (Fig. 3g; geodesic: E1 and E2: *P* < 0.0001; E3 and E4: *P* < 0.0001; E5 and E6: *P* = 0.0001; POST: *P* = 0.0295; Euclidean: E1 and E2: *P* = 0.0094; E3 and E4: *P* = 0.0094; E5 and E6: *P* = 0.034; POST: *P* = 0.0825; differences from PRE; 10,000 permutations; FDR-corrected;  Methods). Remarkably, the patients who developed a robust hippocampal–entorhinal successor representation showed longer reaction times during trials in POST that violated the pyramid rules from exposure phases (see ‘Individual neurons’), which suggests that this representation was used to guide behaviour (Extended Data Fig. 1c). A control analysis performed on neurons outside of the hippocampal–entorhinal complex did not show any significant evidence that the pyramid representation was present during POST, either when compared to the geodesic, Euclidean or successor templates (Extended Data Fig. 6b). Together, these findings demonstrate that the coordinated activity of multiple hippocampal–entorhinal neurons progressively represented a detailed structure of the entire temporal structure and that this representation was predictive in nature.

**Fig. 4:**
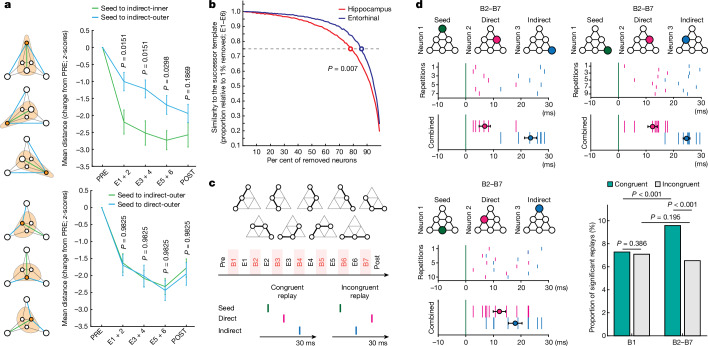
Modulation of receptive fields, regional differences and neuronal replay. **a**, Top, selective hippocampal–entorhinal neurons that preferred a stimulus at an outer node (*n* = 119) responded significantly differently to stimuli from indirect-inner versus indirect-outer nodes, suggesting that these neurons’ receptive fields progressively elongated. Bottom, there was no such effect for neurons that preferred a stimulus at an inner node (*n* = 144), which suggests that their receptive fields were rather symmetrical. Plots show the mean Euclidean distance (±s.e.m.) between responses to respective stimuli (data centred on PRE and *z*-scored per neuron). *P* values from Wilcoxon rank-sum tests (two-sided, FDR-corrected). Orange circles indicate the locations of preferred stimuli. Orange areas illustrate the hypothesized shapes of receptive fields. **b**, The successor representation in the hippocampus was more impaired by growing proportions of artificially removed neurons than in the entorhinal cortex. Similarity to the successor template is plotted as a function of the percentage of removed neurons (relative to 1% of neurons removed; for each 1% step, we randomly selected a given proportion of neurons 10,000 times). The actual difference between the third quartiles was compared with the same difference in 1,000 permutations of the region labels. **c**, The replay analysis focused on three-element graph trajectories consisting of one seed node, a direct node and an indirect node. We analysed triplets of selective hippocampal–entorhinal neurons (recorded in the same session) whose preferred stimuli mapped onto those trajectories. Only spiking activity during breaks between phases was analysed (B1–B7). **d**, Examples of pyramid-congruent replays detected for triplets of selective hippocampal–entorhinal neurons. Coloured circles indicate the graph location of each neuron’s preferred stimulus during PRE. Raster plots show the spiking activity of ‘direct’ and ‘indirect’ neurons during each spontaneous repetition of a given replay. The bottom plot shows combined spiking activity across all repetitions and the mean spikes’ latencies (±s.e.m.). Plots are time-locked to the seed neuron’s relevant spikes. The probability of pyramid-congruent replays increased throughout the study and in B2–B7 was significantly higher than that of incongruent replays (1,000 random permutations of ‘direct’ and ‘indirect’ spike labels). *P* values in **b**,**d** were calculated as the number of permutations with a higher difference than the one actually detected, divided by the total number of permutations. If in none of the permutations the difference was above the actual one, the *P* < 0.001 range is reported. No adjustment for multiple comparisons was applied in **d**.

**Extended Data Fig. 7:**
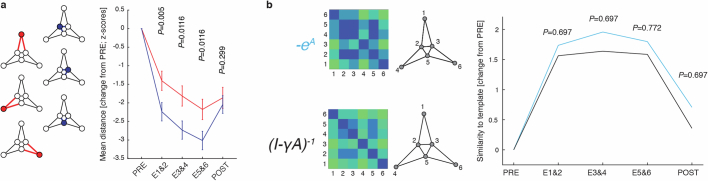
Additional support for the successor model and a comparison of different successor representation metrics. (**a**) An important prediction of the successor representation, but not the geodesic representation, is that direct links between inner nodes (inner-inner) should be shorter than direct links between each outer node and the adjacent inner nodes (outer-inner). We calculated pairwise distances between spiking responses of all selective hippocampal-entorhinal neurons grouped by their preferred node. Then, we compared the mean distances of inner-inner links (2-3, 3-5, 5-2) to the mean distance of outer-inner links (4-2, 4-5, 6-5, 6-3, 1-2, 1-3). As predicted, the former were significantly shorter than the latter (*P* values from Wilcoxon rank-sum tests; two-sided; FDR-corrected). The plot shows mean distances from all 263 (outer: *n* = 119; inner: *n* = 144) selective hippocampal-entorhinal neurons ± s.e.m. (**b**) There were no significant differences between the two measures of the successor representation in terms of similarity to the neuronal data. The plot shows distance matrixes and graphs corresponding to the two measures (see Methods). Each graph shows the most faithful 2D representations of the respective distance matrix obtained from the multidimensional scaling analysis. The right panel shows the degree of similarity between data (546 hippocampal-entorhinal neurons) and each template throughout the study (Spearman’s correlation coefficients; Fisher-transformed; change from PRE). *P* values (FDR-corrected) were calculated as the number of permutations with a higher difference between the templates than the one actually detected, divided by the total number of permutations (10,000).

Apart from affecting the pyramid encoding at the population level (see above), the successor representation should modulate the activity of individual neurons. For example, during spatial navigation, the successor model accounts for the warping of place cells’ receptive fields around environmental barriers^17^. If the pyramid representation involved similar mechanisms, the receptive fields of neurons representing the pyramid’s outer nodes should elongate throughout the study because, from these nodes, the ‘agent’ can only proceed in one general direction (that is, back). Conversely, receptive fields of neurons representing the inner nodes of the pyramid should be more symmetric, as from these nodes, the agent can move in three directions. To complement our neuronal population results that support the above hypotheses (Fig. 3e), we measured the distance between individual neurons’ responses to different stimuli. By analogy with place cells, we analysed selective hippocampal–entorhinal neurons grouped by their preferred node (inner: *n* = 144; outer: *n* = 119). We found that neurons selective to an outer node responded significantly differently to indirect-inner versus indirect-outer nodes, which is consistent with the elongation of their receptive fields (Fig. 4a; E1 and E2: *P* = 0.0151; E3 and E4: *P* = 0.0151; E5 and E6: *P* = 0.0298; POST: *P* = 0.1869; two-sided Wilcoxon rank-sum test; FDR-corrected). By contrast, neurons preferring an inner node did not respond significantly differently to various outer nodes, which suggests that their receptive fields remained symmetric (Fig. 4a; *P* = 0.9825 in all phases; two-sided Wilcoxon rank-sum test, FDR-corrected). Additionally, we found that the ‘inner-to-inner distances’ became shorter than the ‘outer-to-inner distances’, which is also in line with the successor representation (Extended Data Fig. 7a; E1 and E2: *P* = 0.005; E3 and E4: *P* = 0.0116; E5 and E6: *P* = 0.0116; POST: *P* = 0.299; two-sided Wilcoxon rank-sum test; FDR-corrected). The above results closely resemble functional properties of place cells during spatial navigation and reveal single-neuron mechanisms of predictive representations of temporal structures.

## Hippocampal versus entorhinal codes

Next, we tested whether the neuronal pyramid representation differed between the hippocampus and the entorhinal cortex. We found that during exposure phases (E1–E6), hippocampal neurons represented the pyramid more accurately than entorhinal neurons (successor: *P* = 0.0429; Euclidean: *P* = 0.0055; geodesic: *P* = 0.0042; H minus EC difference between Spearman correlation coefficients for each template; *P* values based on 10,000 permutations of the brain region labels). The above result is not simply due to a different number of hippocampal and entorhinal neurons that we detected in this study, as the above analysis balanced this aspect (10,000 random selections of subsets of hippocampal neurons to match the number of entorhinal neurons). Interestingly, during POST, the successor representation was more preserved in the entorhinal cortex than in the hippocampus, suggesting that the former utilizes a more stable neuronal code than the latter (successor: *P* = 0.037; Euclidean: *P* = 0.5963; geodesic: *P* = 0.5484; EC minus H difference between Spearman correlation coefficients for each template; *P* values based on 10,000 permutations of region labels; the number of neurons was balanced; see above). We also tested the robustness of hippocampal versus entorhinal representations against removing growing proportions of neurons from each region. The presumably more structural (‘pure-position’) neural code in the entorhinal cortex should be less affected by such removals than the relational (object-based) code in the hippocampus^2,7^. Indeed, as we removed more neurons from the analysis, similarity to the successor template diminished more rapidly in the hippocampus than in the entorhinal cortex (Fig. 4b; *P* = 0.007; difference between third quartiles; *P* value from 1,000 permutations of region labels; number of neurons balanced; combined data from E1–E6). Analogous differences were not significant for the Euclidean and geodesic templates (*P* = 0.622 and *P* = 0.296, respectively). The above findings suggest that the hippocampus contains a more dynamic object-related representation of temporal sequences, whereas the entorhinal cortex uses a more stable structural code.

## Neuronal replay

Neuronal representation of the pyramid was likely to rely on mechanisms of synaptic plasticity, where the ordering of spikes from the pre-and post-synaptic cells determines whether long-term potentiation or depression occurs^24^. But how can relations between stimuli that occurred seconds apart rely on synaptic phenomena that have a time window of approximately 30ms? One possible explanation is neuronal replay, which refers to a time-compressed reactivation of experienced place cell sequences happening during rest or sleep^25–27^. Whether an analogous single-neuron mechanism exists in humans during the encoding of non-spatial relations remains largely unknown. We looked for triplets of selective hippocampal–entorhinal neurons whose preferred stimuli mapped onto three-node trajectories experienced during exposure phases (Fig. 4c). Each triplet consisted of a neuron selective to an image (‘seed neuron’), a second neuron selective to a directly linked image (‘direct neuron’), and a third neuron that was selective to an indirectly linked image (‘indirect neuron’). Putative replays were defined as consistent firing of the direct and indirect neurons within 30 ms after the seed neuron’s spike. In pyramid-congruent replays, the direct neuron should fire before the indirect one. By contrast, during incongruent replays, which we used as a control condition, the indirect neuron would fire first (Fig. 4c). Importantly, this analysis used only data recorded during breaks (B1, a break after PRE; B2–B7, breaks after each exposure phase). We found that the proportion of congruent replays significantly increased during the course of learning, whereas the proportion of incongruent replays did not change significantly (Fig. 4d; congruent: *P* < 0.001; incongruent: *P* = 0.195; 1,000 permutations of the ‘direct’ and ‘indirect’ spike labels). The above findings bridge the gap between behavioural and synaptic timescales and demonstrate that the neural representations of spatial and temporal structures rely on similar neurophysiological mechanisms.

## Discussion

The human experience is the integration of events characterized by objects with spatial and temporal coordinates—the ‘what’, ‘where’ and ‘when’ of information processing performed by the brain. In the present study, we examined the neural integration of the ‘what’ and ‘when’ of human experience to encode the underlying temporal structure of events. We find that such integration is a process explicitly expressed in the activity of neurons in the hippocampal–entorhinal system, albeit largely implicitly by participant’s awareness. Responses of these neurons scaled with distances between respective nodes of the spatiotemporal graph, thus reflecting the relational contingencies between events characterizing the experience and enabling the predictive representation of expected future states. This neuronal ensemble developed relatively rapidly during the study and remained even when the temporal structure was no longer present. The pyramid graph was extracted directly from experience, without explicitly instructing the participants, and it was abstracted away from the specifics of the task, such as image orientation or behavioural responses.

Our findings provide important insights into the fundamental question of how the human brain forms temporal associations, a critical component in the encoding of episodic memories. Only recently, studies have begun to reveal how this process is implemented by individual neurons in the human MTL. It was demonstrated that cells initially responding only to the picture of a given person started firing to the picture of a given place as a result of the experimental simultaneous pairing of the ‘what’ and ‘where’^28^. It was also shown that the degree of subjectively reported association between two objects could be successfully predicted from the neurons’ responses^29^. The above evidence, combined with results from animal studies^30^, suggests that the MTL has a critical role in the encoding of relational knowledge^31^. The present study extends this view by demonstrating that hippocampal–entorhinal neurons dynamically embed a complex matrix of ‘what’ and ‘when’ contingencies, by precisely scaling their firing rates to the temporal distance between events during sequential experience.

The present study is also in line with the idea that the hippocampal–entorhinal system is critically involved in the abstraction of knowledge. Such abstraction has been described as a cognitive map in the context of spatial navigation^6,7^ and ‘schemas’ or ‘learning sets’ in the context of human behaviour and memory research^32,33^. Recent computational research suggests that the brain implements similar neural mechanisms to extract the underlying structure of spatial as well as non-spatial problems and that the integration of ‘what’, ‘where’ and ‘when’ is essential for this process^7,34,35^. The temporal relational neurons that we identify here in human participants during a non-spatial task, have important implications for the hippocampal–entorhinal system as a neural substrate of the cognitive map.

Arguably the main purpose of extracting the underlying structure of temporal sequences is to predict what is likely to happen next in order to choose appropriate actions and maximize reward^17–20^. A recent computational study showed that neuronal firing patterns that are classically attributed to the encoding of space, such as place cells and grid cells, can be modelled using a predictive successor representation of likely future states, which accounted for a range of empirical findings that cannot be explained by purely Euclidean or geodesic representations^17^. Furthermore, the successor representation can be simulated with neural phenomena that are known to exist in the hippocampal–entorhinal formation, such as the theta phase precession and spike-timing dependent plasticity^20^. Our finding that the neuronal representation of the pyramid graph resembled the successor representation provides the human single-neuron evidence supporting the predictive nature of the hippocampal–entorhinal system function.

The human single-neuron methodology implemented in this study provided a unique window into the possible mechanisms by which the neuronal reorganization occurred during a temporally structured experience. One such mechanism that we demonstrate here is the experience-dependent replay of neuronal firing of specific hippocampal–entorhinal cells taking place between experiment phases. These findings extend previous evidence from rodent studies by showing that encoding of temporal relations between abstract objects in humans engages mechanisms similar to the encoding of spatial trajectories^25–27,36^. These results also expand existing evidence from human studies in which replay has been tested more indirectly, by comparing general patterns of neural activity during and after a given experience^37–41^ or by detecting ‘sharp-wave ripples’ that in rodents often co-occur with replay of individual neurons^42^.

In this study, the neural pyramid topology developed spontaneously from the mere observation of a temporal sequence, without the participants’ detailed explicit knowledge of existent regularity. This finding is consistent with a growing body of evidence that the MTL has a key role in the implicit learning of statistical patterns which does not require deliberate intention or cognitive effort^1,43–45^. For example, a recent study^45^ using human intracranial electroencephalography found that early cortical processing tracked individual syllables, whereas the hippocampus encoded the ordinal position and identity of pseudowords. The present study demonstrates how individual neurons in the human hippocampal–entorhinal system may encode such implicit structure of temporal associations between serial elements of information.

**Extended Data Fig. 8:**
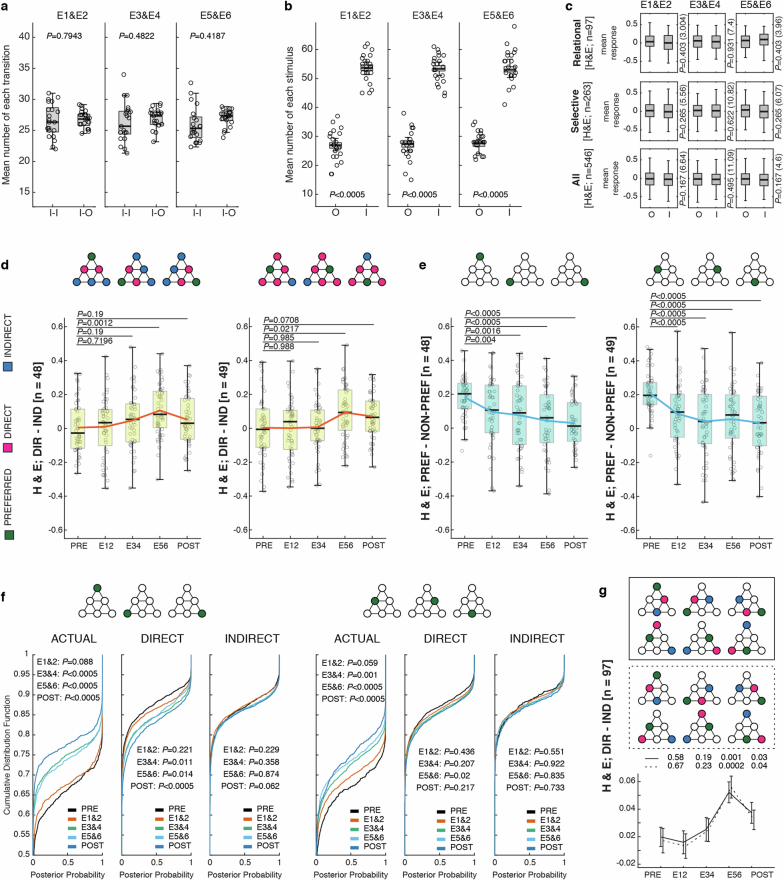
Stimulus familiarity. (**a**) The mean number of outer-inner or inner-outer (I-O) transitions did not differ significantly from the mean number of inner-inner (I-I) transitions (*P* values from the Wilcoxon signed-rank tests; two-sided; FDR-corrected). (**b**) Images from the inner nodes were displayed more frequently during exposure phases than images from the outer nodes (*P* values from the Wilcoxon signed-rank tests; two-sided; FDR-corrected; exact *P* values equal to 5.88 × 10^−5^ for all comparisons). (**c**) Hippocampal-entorhinal neurons did not respond significantly differently to images from the inner versus outer nodes (Wilcoxon signed-rank tests; two-sided; FDR-corrected). Bayes factors supported the null hypotheses (BF_01_) and are provided in the brackets (paired t-tests; two-sided; Cauchy prior; 0.7071). (**d**) Relational neurons responded more strongly to directly linked stimuli, regardless of whether their preferred stimulus was at an inner or an outer node (see Extended Data Fig. 4; Wilcoxon signed-rank tests against PRE; two-sided; FDR-corrected). (**e**) These neurons gradually diminished their selectivity, regardless of whether their preferred stimulus was at an inner or an outer node (see Extended Data Fig. 4; Wilcoxon signed-rank tests against PRE; two-sided; FDR-corrected; exact *P* values for ‘outer’ (from the bottom to the top): *P* = 0.004; *P* = 0.016; *P* = 2.74 × 10^−5^; *P* = 1.73 × 10^−5^; exact *P* values for ‘inner’(from the bottom to the top): *P* = 0.00014; *P* = 6.56 × 10^−5^; *P* = 9.43 × 10^−5^; *P* = 5.65 × 10^−5^). There were no significant differences between the slopes of diminishing selectivity for relational neurons preferring the inner versus outer nodes (outer versus inner; PRE: *P* = 0.566; E1&2: *P* = 0.637; E3&4: *P* = 0.514; E5&6: *P* = 0.831; POST: *P* = 0.86; Wilcoxon signed-rank tests, two-sided). (**f**) We replicated our decoding results (see Fig. 3c) regardless of whether the ‘actual’ image was at an inner or an outer node. Plots show data from all hippocampal-entorhinal neurons (*n* = 546). *P* values from Kolmogorov-Smirnov tests between CDFs of respective posterior probabilities (PRE versus subsequent study phases; one-sided). Please note that data used for training (PRE) contained the same number of repetitions of each image. Exact *P* values for ‘outer-actual’ E3&4: *P* = 1.56 × 10^−8^; E5&6: *P* = 4.82 × 10^−8^; POST: *P* = 4.82 × 10^−13^. Exact *P*-value for ‘outer-direct’ POST: *P* = 6.08 × 10^−6^. Exact *P* values for ‘inner-actual’ E5&6: *P* = 2.72 × 10^−6^; POST: *P* = 1.58 × 10^−4^. (**g**) We also analysed responses of relational neurons when direct and indirect stimuli were repeated a similar number of times (i.e., direct-inner versus indirect-inner; direct-outer versus indirect-outer; all possible combinations). We found the same pattern of results as before (*P* values from Wilcoxon signed-rank tests against PRE; one-sided). The plot shows the mean ‘direct minus indirect’ difference ( ±s.e.m.) in the 0.1 to 1 s time window (peak-normalized and baseline-corrected). For the definition of box plots, please see the legend of Extended Data Fig. 1.

The probabilities of inner-inner and outer-inner node transitions did not differ significantly, so there is no reason to assume that the transition rates determined the strength of respective associations (Extended Data Fig. 8a). However, the inner nodes were presented more frequently during exposure phases than the outer nodes (Extended Data Fig. 8b). This is a natural consequence of the pyramid structure combined with a random walk policy, which happens to mimic many real-life situations (for example, central hubs of a metro system are visited more often than peripheral ones) and experimental setups (for example, a T-maze). However, one could argue that some neurons gradually increased or decreased their firing rate simply owing to stimulus familiarity, which would affect the neural distances between respective nodes. We found that neither relational, selective nor all detected hippocampal–entorhinal neurons responded significantly differently to the inner versus outer nodes (Bayes factors supported the null hypotheses; Extended Data Fig. 8c). In fact, the proportion of hippocampal–entorhinal neurons that significantly increased or decreased their responses to the inner or outer nodes did not significantly differ from chance level (that is, we analysed responses of each hippocampal–entorhinal neuron to all inner or all outer nodes in E5 and E6 versus E1 and E2; *n* = 10 and *n* = 6, respectively; 2% and 1% of all hippocampal–entorhinal neurons, respectively; *P* > 0.99 and *P* = 0.967, respectively; analysis analogous to the Extended Data Fig. 2). Furthermore, we replicated all principal findings of this study when the analysis included only inner or only outer nodes (Extended Data Fig. 8d–g). Thus, stimulus familiarity did not drive our main results. Future studies focusing on how different transition strategies affect the geometry of neuronal representations could manipulate this aspect by using a random walk versus Hamiltonian cycles or other policies.

**Extended Data Fig. 9:**
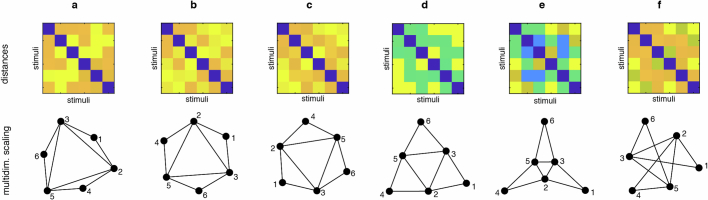
A simulation study supports that a reconstruction of a successor-like pyramid representation requires distance-dependent scaling of neural responses rather than simple pairwise associations. Please note the overall similarity between panel e above and the pyramid reconstruction from the neuronal data (Fig. 3f). For each of the following scenarios (a-f), we generated 6,000 artificial neurons. Each neuron could respond preferentially to any of the six stimuli (random assignment) and this response could vary between 1 to 15 Hz (random assignment). Then, we calculated Euclidean distances between responses to each pair of stimuli across all neurons and used multidimensional scaling to provide the most faithful reconstruction of the respective matrix in 2D (see Methods). (**a**) Neurons respond to the preferred stimulus and only one directly linked stimulus on the pyramid; additionally, responses to ‘direct’ are not scaled (i.e., any value between 1 and 15 Hz). (**b**) Neurons respond to the preferred stimulus and only one directly linked stimulus; responses to ‘direct’ are scaled (i.e., 50% of the response to the preferred stimulus). (**c**) Neurons respond to the preferred stimulus and all directly linked stimuli; responses to ‘direct’ are not scaled (see earlier). (**d**) Neurons respond to the preferred stimulus and all directly linked stimuli; responses to ‘direct’ are scaled (i.e., 50% of the response to preferred stimulus). (**e**) Neurons respond to the preferred stimulus and all directly linked stimuli; responses are scaled differently for outer and inner nodes (percentages relative to responses to the preferred stimulus; outer-seeds: direct – 40%; indirect-inner – 20%; indirect-outer – 0%; inner-seeds: direct-inner – 80%; direct-outer – 60%; indirect – 60%). (**f**) Neurons respond to the preferred stimulus and only one stimulus of each type (outer-seeds: direct – 40%; indirect-inner – 20%; indirect-outer – 0%; inner: direct-inner – 80%; direct-outer – 60%; indirect – 60%).

**Extended Data Fig. 10:**
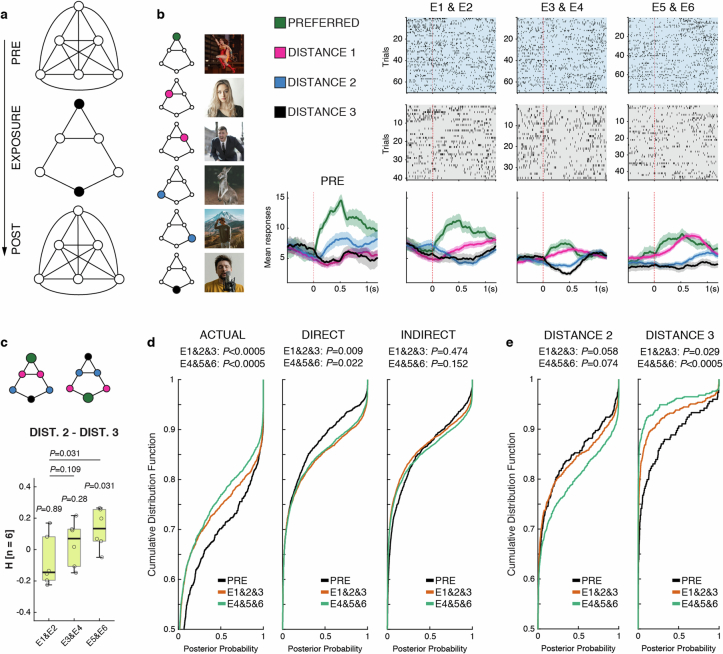
Hippocampal neurons encoded another complex temporal structure with longer paths. (**a**) In a separate study, we tested five additional patients (seven recording sessions; see Methods). The procedure was the same as before, but we removed two edges from the pyramid graph during exposure. The resulting ‘diamond’ structure had the ‘shortest path’ distances of length one (D1; corresponding to the ‘direct’ category in the main study) or lengths two and three (D2 and D3, respectively, corresponding to the ‘indirect’ category in the main study). We recorded 221 neurons, of which 55 were located in the hippocampus (we did not have any recording sites in the entorhinal cortex in this additional study). Six hippocampal neurons responded preferentially during PRE to stimuli at the most distant nodes of the diamond (black circles) and were relational neurons according to the criteria described earlier (see Methods). (**b**) A relational neuron from the left hippocampus showing progressive tuning to the graph’s distances. Raster plots show individual spikes during each stimulus presentation in E1&2, E3&4, and E5&6. Line plots show the mean number of spikes ± s.e.m. (PRE included for reference). The left panel shows the stimuli and their locations on the graph. (**c**) During late-exposure phases, these hippocampal relational neurons responded more strongly to images located two edges away from their preferred stimulus than to images located three edges away (Wilcoxon signed-rank tests against E1&2 or zero; one-sided). Each circle corresponds to one neuron (the average D2 minus D3 difference in the 0.1 to 1.3 s time window, peak-normalized and baseline-corrected). For the definition of box plots, please see the legend of the Extended Data Fig. 1. (**d**) We replicated the main population decoding finding that neuronal representations of adjacent nodes progressively overlapped (see Fig. 3c). That is, the probability of decoding the actual stimulus as ‘actual’ was gradually decreasing, the probability of decoding the direct stimuli as ‘actual’ was increasing, and the probability of decoding indirect stimuli as ‘actual’ did not change significantly. This analysis was conducted on all hippocampal neurons from this additional study (*n* = 55) during all stimuli presentations. Please note that the indirect category combines D2 and D3. *P* values obtained from Kolmogorov-Smirnov tests comparing CDFs in PRE versus the subsequent study phases (one-sided). Exact *P* values for ‘actual’ E1&2&3: *P* = 1.06 × 10^−7^; E4&5&6: *P* = 1.94 × 10^−7^. (**e**) Next, we analysed CDFs for nodes separated by two versus three links away from the actual stimulus. This analysis was conducted only for trials where the actual stimulus was located at one of the ‘black nodes’ of the diamond (see panel a). We found that the probability of decoding D2 stimuli as ‘actual’ gradually increased during the study, while the probability of decoding D3 stimuli as ‘actual’ gradually decreased. *P* values obtained from Kolmogorov-Smirnov tests (two-sided). Exact *P*-value for ‘distance 3’ E4&5&6: *P* = 3.69 × 10^−6^.

One might ask whether the current design allows us to disambiguate between distance-dependent scaling and the formation of simple pairwise associations, since every pair of nodes that was not a direct link on the pyramid automatically was two links apart. However, if multiple respective links were not scaled according to a common metric (distance), it would not be possible to recover the entire pyramid graph from the neuronal population activity, especially not the successor representation where various direct and indirect links have different lengths (Extended Data Fig. 9). Such a reconstruction was possible in the present study (Fig. 3f). To further address this point, we collected data from five additional patients (7 sessions; 221 neurons) with a diamond-shaped graph where links to indirect stimuli varied between two and three edges (D2 and D3, respectively). We found that, during late-exposure phases, hippocampal relational neurons responded more strongly to images located two edges away from their preferred stimulus than to images located three edges away. We also replicated population decoding results from the main study and showed that the representational overlap was greater for D2 stimuli than for D3 (Extended Data Fig. 10). The above evidence supports distance-dependent scaling in the encoding of the temporal structure of the sequence.

Together, the findings of this study reveal multiple similarities between the neurophysiological properties of individual cells representing locations in physical space and neurons encoding abstract objects in a temporal sequence structure; these parallels include reorganization and functional overlap of representations of adjacent states, experience-dependent and predictive modulation of receptive fields, as well as offline replay of individual neurons’ activity congruent with past experience. Thus, the human brain appears to be using analogous mechanisms to represent seemingly very different types of information: relations in space and time. The remarkable entorhinal–hippocampal neuronal machinery likely evolved to form scalable and partly non-Euclidean (‘warped’) representations of space-time trajectories to enable learning and prediction, necessary for the organism’s survival. Here, keeping space constant, we demonstrate at the neuronal level how such representations of object trajectories in time are incorporated by the human entorhinal–hippocampal system.

## Methods

### Participants

The participants were 17 patients with intractable epilepsy who were implanted with depth electrodes to delineate a potentially surgically treatable epileptogenetic zone. Demographics information and neuropsychological scores are presented in Supplementary Table 1. Electrode placements were determined solely on the basis of clinical treatment criteria. The follow-up studies (Extended Data Figs. 1 and 10) included 33 healthy controls (26 female participants; mean age: 31 ± 7 years old) and 5 additional patients with epilepsy (2 female participants; mean age: 38 ± 12 years old). All participants volunteered for the study by providing informed consent according to a protocol approved by the UCLA Medical Institutional Review Board (IRB).

### Neural recordings

Patients were stereotactically implanted with 7–12 Behnke-Fried electrodes with 40-µm diameter microwire extensions (eight high-impedance recording wires and one low-impedance reference wire per depth electrode) that capture local field potentials and extracellular spike waveforms^46^. Microwire electrophysiology data were amplified and recorded at 30 kHz on a Blackrock Microsystems recording system or at 32 kHz on a Neuralynx recording system (Cheetah 5.0).

### Microelectrode localizations

Prior to data collection, each microelectrode location was confirmed by an expert neurosurgeon (I.F.) based on the patient’s postoperative computed tomography (CT) scan with visible electrode artifacts overlaid on a co-registered preoperative T1 structural MRI (BrainLab software). For descriptive purposes (Fig. 1d), we additionally used the following procedure to transform locations from each participant’s ‘native brain space’ to the standard Montreal Neurological Institute (MNI) space. First, each participant’s MRI and CT images were co-registered using the FSL ‘flirt’ function. Second, the MRI image was: (1) segmented into the grey matter, white matter, and cerebrospinal fluid probability maps; (2) resampled (1 × 1 × 1 mm voxel size); and (3) normalized to the 152 T1-weighted MNI template using the nonlinear transformation algorithm implemented in the Statistical Parametric Mapping toolbox (SPM12, Wellcome Department of Cognitive Neurology, London, UK). Third, the same transformation parameters were applied to the participant’s CT image. MNI coordinates for each microelectrode were extracted manually from the normalized CT overlaid on the normalized MRI from a given participant using the FSLeyes software.

### General procedure

Before the main experiment (typically 1–2 days prior), a screening experiment was conducted to find 6 stimuli (images of people) associated with robust and preferential responses of single neurons in the MTL. These six images were then used during the main experimental task (Fig. 1b), which was introduced to the patients as a follow-up of the screening study without mentioning that the stimuli would be presented in a specific order. At the end of the main experiment, we asked the participants to answer the following questions: “Have you noticed any pattern in the sequence of images shown in any of the phases? If yes, what was it?”; “Have you had any special strategy during this study?”. None of the participants reported noticing any pattern that was relevant to the experimental manipulation (Fig. 1b). We used the Psychophysics Toolbox to control the timings of stimuli presentation and register behavioural responses^47^.

### Screening session

During screening, approximately 120 images were repeatedly shown to the patients on a laptop computer (taking around 40 min). These images showed people, animals, objects and landmarks that were partly selected based on the participant’s preferences (for example, favourite actors, musicians, places, etc.). The experiment consisted of eight blocks, each with a different instruction (for example, block 1: “Determine whether each image shows a person or not”; block 2: “Determine whether each image shows a plant or not”; etc.). Each image was presented exactly once during each block, for the duration of 1 s, against a black background. The order of stimuli presentation was random. Participants indicated their responses using two assigned keys on a hand-held game pad.

### Experimental task

The main study consisted of three parts: pre-exposure (PRE), exposure (E1–E6), and post-exposure (POST; Fig. 1c). During PRE (121 stimuli presented), all images were displayed in a pseudo random sequence (60 direct and 60 indirect graph-transitions; on average, each direct transition was presented 7 times and each indirect transition 9 times). The task was to determine whether each image showed a male or female (gender task). The participants used the right and left arrow keys on a laptop keyboard to indicate their responses. During the six subsequent exposure phases (121 trials in each phase), the order of stimuli was still randomized but restricted by the topological structure of the pyramid graph (Fig. 1c) so that only images directly linked on the graph were shown immediately after another. The starting location was selected randomly in each experiment phase. The behavioural task during all exposure phases was to determine whether a given image was mirrored or not when compared to PRE (Fig. 1c; the participants used the right and left arrow keys on a laptop keyboard to indicate their responses). During each phase, 61 images were ‘normal’ and 60 were ‘mirrored’. The order of mirrored and normal images was random. The POST phase was the same as PRE (all stimuli presented in a pseudo random sequence, without the ‘pyramid rule’; on average, each direct transition was presented 7 times and each indirect transition 8 times). Behavioural instructions displayed in the beginning of each phase emphasized that the participants should try to respond as quickly and accurately as possible. The first trial in each phase (that is, the beginning of a sequence) was discarded from the analyses, so effectively each phase consisted of 120 trials. The experiment in all phases was self-paced, that is: (1) a given image was displayed for as long as it took the participant to respond; and (2) the participants could have had breaks between phases for as long as they needed. All stimuli were displayed against a grey background. During a randomized inter-trial interval (1-3 s), a black ‘fixation’ circle was displayed in the middle of the screen. After each stimulus presentation, the participants received feedback (“correct!” or “incorrect” in relation to the currently performed task) displayed for 500 ms. All trials (correct and incorrect) were included in the analysis of electrophysiological data, as the behavioural tasks were unrelated to the main research question. Behavioural accuracy of responses during PRE and POST was near-perfect indicating that the gender task was easy for all the participants (Extended Data Fig. 1a). Accuracy in the ‘mirror task’ was lower but improved over the course of the study (Extended Data Fig. 1a; this task was supposed to be more challenging to maintain the participants’ attention).

### Spike sorting

Automated spike detection and sorting were performed using the WaveClus3 software package in MATLAB^48^. We then manually reviewed each unit for inclusion by evaluating the waveform’s shape, amplitude, inter-spike intervals, and firing consistency across study phases. We rejected units that were likely contaminated by artifacts, in keeping with field-standard spike evaluation criteria^49^. For electrodes with multiple putative units that passed this inclusion check, we merged units whose waveform features could not be well-separated in principal components space, retaining for analysis a combination of single- and multi-units.

### Single-neuron analyses

For each neuron and each stimulus presentation, we selected a time window around the stimulus onset (from −1 to +2 s). Then we calculated the number of spikes in 0.1 s time bins, smoothed (moving sum: ± 0.25 s) and baseline-corrected the data (subtracted the mean activity in the −0.5 to 0 s time window). The ‘response window’ was defined from 0.1 to 1.2 s after the stimulus onset. For a given neuron, the ‘preferred stimulus’ was the image associated with the strongest mean response in the response window during PRE. Depending on the position of the preferred stimulus on the pyramid, the remaining images were labelled as ‘direct’ or ‘indirect’ (Fig. 1b). This assignment was used across all study phases. ‘Selective neurons’ were defined as cells that during PRE: (1) responded significantly stronger to the preferred stimulus in the response window versus baseline; and (2) responded significantly stronger to the preferred stimulus than to the remaining stimuli combined. ‘Relational neurons’ were defined as cells that: (1) were selective (see above); (2) responded significantly stronger to the direct than indirect stimuli during E5 and E6; and (3) responded significantly stronger to direct stimuli during E5 and E6 than during E1 and E2. The ‘diminishing selectivity neurons’ were defined as cells that: (1) were selective; and (2) responded significantly weaker to the preferred stimulus in E5 and E6 than in E1 and E2. All the above criteria were tested with the Wilcoxon signed-rank tests (one-sided) with a *P* value threshold of 0.05. The above procedure was repeated 1,000 times, with random permutations of the stimulus or phase labels, depending on which criterion was tested. These permutations informed how many neurons of a given type are expected in a given brain region by chance. The empirical *P* value was calculated as the number of permutations with more neurons of a given type than the number of neurons actually detected, divided by the total number of permutations. If this value was less than 0.05, we concluded that a given brain region contained a significant proportion of a given neuron type (Extended Data Fig. 2 and Supplementary Table 3). To analyse combined responses of all relational neurons (Extended Data Fig. 4), we calculated the difference between each neuron’s mean responses to direct minus indirect stimuli and preferred minus non-preferred stimuli. This was done for each study phase separately. Then, we peak-normalized and baseline-corrected (−0.5 to 0 s) those differences and extracted the mean from the 0.1 to 1 s time window. For line plots showing the mean responses of individual neurons (Fig. 2a,b and Extended Data Figs. 3a and 9b), we used 0.01 s bins and the ±0.25 s moving sum. For plots showing multiple neurons (Figs. 1f and 2c), we *z*-scored and baseline-corrected (−0.5 to 0 s) the data from each neuron (for illustration purposes, we used the ± 0.2 s moving sum and heat maps were additionally smoothed with ± 0.1 s moving average).

### Neural population analyses

To decode stimulus identity during each image presentation, we used the Poisson naive Bayes classifier, as implemented in the Neural Decoding Toolbox^50^. The spiking activity of each neuron was extracted from the −1 to +2 s time window relative to the stimulus onset. Data was binned (0.1 s) and smoothed (moving sum: ±0.25 s). The decoder was run on the summed spiking activity in the 0.1 to 1 s time window (Extended Data Fig. 5). The main analysis focused on posterior probabilities assigned by the decoder to the image actually presented (actual), images directly linked to that stimulus on the graph (direct), and images linked indirectly (indirect). The analysis was performed for each recording session separately (different stimuli), but the resulting probability distributions were combined across all sessions and image presentations. The classifier was trained on the data from PRE and tested on all subsequent phases. For testing in PRE, we used the ‘leave-one-trial-out’ cross-validation. Kolmogorov–Smirnov tests were used to compare cumulative distribution functions (CDFs) of posterior probabilities (one-sided). To reconstruct the entire pyramid graph (Fig. 3f,g and Extended Data Figs. 6b and 7b), we calculated Euclidean distances between mean responses of each neuron to each pair of images across all relevant neurons (neurons that stopped firing during the late study phases were excluded; distances were *z*-scored; bin-size: 0.1 s; baseline-correction: −0.5 to 0 s; moving sum: ± 0.15 s; time window: 0.1 to 1 s). Then, we compared the resulting neural distance matrixes to three templates (Fig. 3f). In the geodesic template, distances between each pair of nodes corresponded to the number of edges of the shortest path connecting the nodes. In the Euclidean template, distances between nodes 1–5, 4–3 and 6–2 were calculated from the Pythagorean theorem (right triangles: 1–5–6, 4–3–1, 6–2–1). The remaining distances corresponded to the shortest path (see above). In line with the previous literature^2,51^, the successor template (ST) was calculated as the negative of the matrix exponential of the adjacency matrix *A*:

$${\rm{ST}}={-{\rm{e}}}^{A}=-\mathop{\sum }\limits_{n=0}^{\infty }\frac{{A}^{n}}{n!}$$

The above metric provided a slightly better fit to the data than a related index that defines the relationships between states (Extended Data Fig. 7b):

$$\mathop{\sum }\limits_{n=0}^{\infty }{\gamma }^{n}{A}^{n}={(I-\gamma A)}^{-1}$$

Here, entries *a*_*ij*_ for each *A*^*n*^ correspond to the number of possible paths of length *n* between objects *i* and *j* and a discount factor is 0 < *γ* < 1 (refs. ^2,19^). To illustrate most faithful 2D representations of the respective distance matrixes, we used the multidimensional scaling analysis (MDS; ‘mdscale’ function in MATLAB; criterion: ‘sammon’). Because MDS can only be performed on matrices with positive entries, we normalized the matrixes by adding the absolute value of the matrix’s minimum plus a constant of 0.1. The similarity between neural distance matrixes and each template was calculated as the Spearman correlation (Fisher-transformed). Because the aim was to test how this similarity changes over the course of the study (unconfounded by any potential pre-existing similarity), for each phase, we subtracted the degree of similarity in PRE (Fig. 3g and Extended Data Figs. 6b and 7b). To obtain null distributions of correlation coefficients, the above procedure was repeated 10,000 times with random permutations of the nodes’ positions. *P* values were calculated as the number of permutations with higher correlation coefficients than the one actually detected, divided by the total number of permutations. If in none of the permutations the correlation was above the actual value, the *P* < 0.0001 range is reported.

### Replay analysis

We analysed sessions that contained at least three selective hippocampal–entorhinal neurons from the same hemisphere, whose preferred stimuli from PRE mapped onto three-element pyramid trajectories (Fig. 4c, one seed neuron, one direct neuron and one indirect neuron forming a connected path). For each spike of the seed neuron, we checked whether the direct and indirect neurons fired at least once in the 0 to 30 ms time window. The above situation had to occur at least five times to be included in the analysis (that is, *n* < 5 was considered insufficient for robust statistical inference). There were 536 such putative replays in B1 (break after PRE) and 1,012 in B2–B7 (breaks after exposure phases). If the direct neuron fired significantly earlier than the indirect neuron, the replay was labelled ‘congruent’ (Fig. 4c; we analysed latencies of the first ‘direct spikes’ versus latencies of the first ‘indirect spikes’ across all repetitions of a given replay^52^; Wilcoxon signed-rank test, one-sided, with a *P* value threshold of 0.05). If the opposite was true, a replay was labelled ‘incongruent’. To obtain *P* values for the comparisons between proportions of congruent and incongruent replays throughout the study, we randomly shuffled spikes from the direct and indirect neurons (1,000 permutations) and used the resulting null distribution as reference.

### Reporting summary

Further information on research design is available in the Nature Portfolio Reporting Summary linked to this article.

## Online content

Any methods, additional references, Nature Portfolio reporting summaries, source data, extended data, supplementary information, acknowledgements, peer review information; details of author contributions and competing interests; and statements of data and code availability are available at 10.1038/s41586-024-07973-1.

## Supplementary information

Supplementary Tables This file contains Supplementary Tables 1–6, presenting additional information and results.

Reporting Summary

## Data Availability

Owing to ethical considerations and protection of patients’ confidentiality, data supporting the results of this study are available from the corresponding authors upon a reasonable request—that is, for collaborative research by researchers, adhering to protocols approved by the Institutional Review Board.

## Code availability

Owing to ethical considerations and protection of patients’ confidentiality, code supporting the results of this study is available from the corresponding authors upon a reasonable request—that is, for collaborative research by researchers, adhering to protocols approved by the Institutional Review Board.
