# Supplementary material for: Human hippocampal and entorhinal neurons encode the temporal structure of experience
Source: Nature. 2024 Sep 25;635(8037):160–7. doi: 10.1038/s41586-024-07973-1 (PMC11540853; doi:10.1038/s41586-024-07973-1)
Supplement: Supplementary file 1 — This file contains Supplementary Tables 1–6, presenting additional information and results. [file 41586_2024_7973_MOESM1_ESM.pdf]

nature portfolio

https://doi.org/10.1038/s41586-024-07973-1

Supplementary information

Human hippocampal and entorhinal neurons encode the temporal structure of experience

In the format provided by the authors and unedited

Supplementary Table 1. Participant demographics and neuropsychological scores. ^

ID	Age	Hand.	Sex	Verb. IQ	Perc. IQ	Digit Span	Verbal Memory		Visual Memory	Lang.	Executive Function	
							WMS	*CVLT*			*Trail Making*	*Stroop*
P1	29	R	F	107	92	37	75	16	5	63	42	84
P2	31	R	F	83	121	37	50	69	< 1	14	62	63
P3	24	L	M	105	98	50	63	84	< 1	6	5	84
P4	69	R	M	103 [a]	94 [a]	37	63	50	12	84 [b]	10	12 [c]
P5	50	R	F	107	82	16	16	7	< 1	3	14	84
P6	25	A	F	103 [a]	109 [a]	50	1	2	< 1	18 [b]	37 [d]	75
P7	33	R	F	102	82	63	37	16	79	27	5	16
P8	43	R	F	80	98	16	2	31	3 [e]	2 [b]	4	1 [c]
P9	33	R	F	-	-	25 [f]	91 [f]	93 [f]	21 [f]	70 [f, g]	21[f]	16 [f]
P10	30	R	M	95	109	5	9	70 [f]	50	7 [h]	4 [i]	66 [c]
P11	21	R	F	103	121	63	50	63 [j]	14	81 [k]	81 [i]	75
P12	51	L	M	100	92	37	50	31	16 [e]	66 [b]	58	58 [c]
P13	22	R	M	-	-	-	-	-	-	-	-	-
P14	38	R	F	-	-	-	-	-	-	-	-	-
P15	22	R	M	98	92	16	63 [l]	5 [j]	< 1	37 [k]	3 [i]	16
P16	23	R	M	-	-	-	-	-	-	-	-	-
P17	32	R	F	-	-	-	-	-	-	-	-	-

^Except as noted, Verbal IQ was calculated with the use of the Verbal Comprehension Index score of the Wechsler Adult Intelligence Scale–Fourth Edition (WAIS-IV), Perceptual IQ with the use of the Perceptual Reasoning Index score of WAIS-IV, digit span (i.e., attention) with the use of WAIS-IV, verbal memory by means of the logical memory delayed recall portion of the Wechsler Memory Scale–Fourth Edition (WMS-IV) and the long-delay free-recall portion of the California Verbal Learning Test–Second Edition (CVLT-II), visual memory with the use of the delayed recall trial of the Rey Complex Figure Test, language with the use of Expressive One-Word Picture Vocabulary Test–Fourth Edition (EOWPVT-4), and executive function by means of Trail Making Test Part B and the Delis–Kaplan Executive Function System (D-KEFS) Color-Word Interference Test on the Inhibition trial. Except for Verbal and Perceptual IQ, all scores are given as percentiles. ***Abbreviations:*** *a Prorated score; b Boston Naming Test–Second Edition; c Stroop Color-Word Test (Golden Version) color-word score; d D-KEFS Trail Making Test on the Number-Letter Switching; e 3-minute delayed version of the Rey Complex Figure Test; f testing was performed approximately 3 years prior to phase 2 monitoring; g Neuropsychological Assessment Battery (NAB) Naming Test; h Picture Vocabulary Test of the Woodcock-Johnson IV Tests of Oral Language (WJ-IV-OL); i the oral version of the Trail Making Test Part B (OTMT-B); j the long-delay free-recall portion of the California Verbal Learning Test–Third Edition (CVLT-3); k Expressive Vocabulary Test, Third Edition (EVT-3); l verbal paired associates delayed recall portion of the WMS-IV.*

Supplementary Table 2. Counts of all neurons per session and per region. ^

	A	E	H	I	IT	LT	O	OF	PC	PH	SMA	OTHER	Total:
**P1**	7	21	13	2	0	0	0	7	7	0	2	0	59
**P2**	0	17	18	22	0	0	0	19	9	0	9	8	102
**P3**	0	10	13	0	0	10	18	0	17	12	0	1	81
**P4**	13	0	24	9	0	0	0	27	0	16	0	0	89
**P5**	0	5	4	0	6	4	1	0	0	7	0	0	27
**P6**	9	0	16	0	7	6	0	14	0	5	0	0	57
**P7**	0	19	16	9	0	16	8	0	2	0	0	0	70
**P7**	0	21	16	11	0	32	8	0	7	0	0	0	95
**P8**	0	6	21	0	9	23	0	26	11	0	0	0	96
**P8**	0	2	8	0	4	15	0	14	3	0	0	0	46
**P9**	2	1	8	5	0	0	0	0	2	0	0	10	28
**P9**	7	5	17	21	0	0	0	0	10	0	0	16	76
**P10**	16	11	11	0	0	1	0	13	0	5	0	0	57
**P11**	17	0	11	0	0	0	0	23	0	11	0	0	62
**P12**	14	5	9	0	0	0	0	7	0	3	0	0	38
**P13**	21	26	28	2	0	0	0	11	0	0	0	20	108
**P13**	16	32	20	10	0	0	0	21	0	0	0	19	118
**P14**	31	0	25	0	17	0	0	3	0	0	0	0	76
**P15**	0	21	34	0	0	0	4	0	0	0	8	19	86
**P16**	23	6	11	0	5	0	0	4	0	0	0	0	49
**P17**	3	0	15	0	4	3	0	6	0	0	5	0	36
**Total:**	179	208	338	91	52	110	39	195	68	59	24	93	1456

^*Abbreviations:* A – amygdala; E – entorhinal cortex; H – hippocampus; I – insula and operculum; IT – inferior temporal cortex; LT – lateral temporal cortex; O – occipital cortex; OF – orbitofrontal cortex and anterior cingulate cortex; PC – parietal cortex & middle/posterior cingulate cortex; PH – parahippocampal gyrus; SMA – Supplementary Motor Area.

Supplementary Table 3. Counts of neuron-types. ^

	all identified neurons	selective neurons	relational neurons	diminishing selectivity neurons
A	179	64	10	3
E	208	111***	42*	20***
H	338	152***	55*	31***
I	91	39	13	11***
IT	52	19	5	2
LT	110	58	22	7
O	39	20	11	3
OF	195	66	22	9*
PC	68	24	6	0
PH	59	33***	12	2
SMA	24	12	4	0
OTHER	93	33	12	2
Total:	1456	631	214	90

^*Abbreviations:* A – amygdala; E – entorhinal cortex; H – hippocampus; I – insula and operculum; IT – inferior temporal cortex; LT – lateral temporal cortex; O – occipital cortex; OF – orbitofrontal cortex and anterior cingulate cortex; PC – parietal cortex & middle/posterior cingulate cortex; PH – parahippocampal gyrus; SMA – Supplementary Motor Area. ***Symbols:*** * – P<0.05; ** – P<0.005; *** – P<0.0005. *P*-values calculated as the number of permutations with more neurons of a given type than the number of neurons actually detected, divided by the total number of permutations (i.e., 1,000). For exact *P*-values, please see the Extended Data Fig. 2. No adjustment for multiple comparisons was applied.

Supplementary Table 4. Counts of selective neurons per region and session. ^

	A	E	H	I	IT	LT	O	OF	PC	PH	SMA	OTHER	Total:
**P1**	4	10	5	1	0	0	0	5	1	0	1	0	27
**P2**	0	9	11	16	0	0	0	5	2	0	6	2	51
**P3**	0	6	10	0	0	7	7	0	9	9	0	0	48
**P4**	1	0	16	5	0	0	0	11	0	7	0	0	40
**P5**	0	2	0	0	5	2	1	0	0	4	0	0	14
**P6**	3	0	5	0	1	5	0	7	0	5	0	0	26
**P7**	0	7	4	3	0	10	7	0	2	0	0	0	33
**P7**	0	15	9	5	0	20	2	0	0	0	0	0	51
**P8**	0	3	6	0	3	11	0	14	5	0	0	0	42
**P8**	0	1	4	0	2	3	0	0	2	0	0	0	12
**P9**	1	1	3	2	0	0	0	0	0	0	0	0	7
**P9**	4	2	9	5	0	0	0	0	3	0	0	4	27
**P10**	7	5	0	0	0	0	0	5	0	3	0	0	20
**P11**	4	0	5	0	0	0	0	5	0	4	0	0	18
**P12**	3	2	3	0	0	0	0	2	0	1	0	0	11
**P13**	6	18	5	0	0	0	0	2	0	0	0	12	43
**P13**	6	19	8	2	0	0	0	3	0	0	0	8	46
**P14**	15	0	12	0	5	0	0	3	0	0	0	0	35
**P15**	0	9	27	0	0	0	3	0	0	0	2	7	48
**P16**	8	2	6	0	0	0	0	1	0	0	0	0	17
**P17**	2	0	4	0	3	0	0	3	0	0	3	0	15
**Total:**	64	111	152	39	19	58	20	66	24	33	12	33	631

^*Abbreviations:* A – amygdala; E – entorhinal cortex; H – hippocampus; I – insula and operculum; IT – inferior temporal cortex; LT – lateral temporal cortex; O – occipital cortex; OF – orbitofrontal cortex and anterior cingulate cortex; PC – parietal cortex & middle/posterior cingulate cortex; PH – parahippocampal gyrus; SMA – Supplementary Motor Area.

Supplementary Table 5. Counts of relational neurons per region and session. ^

	A	E	H	I	IT	LT	O	OF	PC	PH	SMA	OTHER	Total:
**P1**	0	2	3	1	0	0	0	0	0	0	1	0	7
**P2**	0	1	3	7	0	0	0	1	2	0	2	0	16
**P3**	0	2	5	0	0	4	6	0	2	3	0	0	22
**P4**	1	0	10	0	0	0	0	4	0	3	0	0	18
**P5**	0	1	0	0	3	0	0	0	0	2	0	0	6
**P6**	0	0	1	0	0	1	0	3	0	2	0	0	7
**P7**	0	3	3	1	0	4	3	0	1	0	0	0	15
**P7**	0	8	3	0	0	9	1	0	0	0	0	0	21
**P8**	0	1	3	0	1	4	0	7	0	0	0	0	16
**P8**	0	0	2	0	0	0	0	0	0	0	0	0	2
**P9**	1	1	2	1	0	0	0	0	0	0	0	0	5
**P9**	1	1	2	2	0	0	0	0	1	0	0	1	8
**P10**	1	2	0	0	0	0	0	0	0	2	0	0	5
**P11**	0	0	0	0	0	0	0	1	0	0	0	0	1
**P12**	0	0	1	0	0	0	0	0	0	0	0	0	1
**P13**	1	6	0	0	0	0	0	1	0	0	0	3	11
**P13**	2	6	3	1	0	0	0	1	0	0	0	3	16
**P14**	2	0	3	0	0	0	0	2	0	0	0	0	7
**P15**	0	7	9	0	0	0	1	0	0	0	1	5	23
**P16**	1	1	1	0	0	0	0	0	0	0	0	0	3
**P17**	0	0	1	0	1	0	0	2	0	0	0	0	4
**Total:**	10	42	55	13	5	22	11	22	6	12	4	12	214

^*Abbreviations:* A – amygdala; E – entorhinal cortex; H – hippocampus; I – insula and operculum; IT – inferior temporal cortex; LT – lateral temporal cortex; O – occipital cortex; OF – orbitofrontal cortex and anterior cingulate cortex; PC – parietal cortex & middle/posterior cingulate cortex; PH – parahippocampal gyrus; SMA – Supplementary Motor Area.

Supplementary Table 6. Counts of diminishing selectivity neurons per region and session. ^

	A	E	H	I	IT	LT	O	OF	PC	PH	SMA	OTHER	Total:
**P1**	0	2	0	0	0	0	0	0	0	0	0	0	2
**P2**	0	3	1	6	0	0	0	1	0	0	0	0	11
**P3**	0	1	5	0	0	1	0	0	0	0	0	0	7
**P4**	0	0	6	0	0	0	0	1	0	2	0	0	9
**P6**	0	0	2	0	0	0	0	2	0	0	0	0	4
**P7**	0	1	1	2	0	1	3	0	0	0	0	0	8
**P7**	0	3	2	2	0	3	0	0	0	0	0	0	10
**P8**	0	0	1	0	0	2	0	3	0	0	0	0	6
**P8**	0	0	0	0	1	0	0	0	0	0	0	0	1
**P9**	1	0	1	0	0	0	0	0	0	0	0	0	2
**P9**	1	0	3	1	0	0	0	0	0	0	0	1	6
**P10**	0	1	0	0	0	0	0	0	0	0	0	0	1
**P11**	0	0	0	0	0	0	0	2	0	0	0	0	2
**P13**	0	3	2	0	0	0	0	0	0	0	0	1	6
**P13**	0	5	1	0	0	0	0	0	0	0	0	0	6
**P15**	0	1	5	0	0	0	0	0	0	0	0	0	6
**P16**	1	0	0	0	0	0	0	0	0	0	0	0	1
**P17**	0	0	1	0	1	0	0	0	0	0	0	0	2
**Total:**	3	20	31	11	2	7	3	9	0	2	0	2	90

^*Abbreviations:* A – amygdala; E – entorhinal cortex; H – hippocampus; I – insula and operculum; IT – inferior temporal cortex; LT – lateral temporal cortex; O – occipital cortex; OF – orbitofrontal cortex and anterior cingulate cortex; PC – parietal cortex & middle/posterior cingulate cortex; PH – parahippocampal gyrus; SMA – Supplementary Motor Area.
