# Supplementary material for: Human hippocampal and entorhinal neurons encode the temporal structure of experience
Source: Nature. 2024 Sep 25;635(8037):160–7. doi: 10.1038/s41586-024-07973-1 (PMC11540853; doi:10.1038/s41586-024-07973-1)
Supplement: Supplementary file 2 — Reporting Summary [file 41586_2024_7973_MOESM2_ESM.pdf]

nature portfolio

2023-05-09324A
Corresponding author(s): Pawel Tacikowski & Itzhak Fried

Last updated by author(s): Jul 10, 2024

Reporting Summary

Nature Portfolio wishes to improve the reproducibility of the work that we publish. This form provides structure for consistency and transparency in reporting. For further information on Nature Portfolio policies, see our Editorial Policies and the Editorial Policy Checklist.

Statistics

For all statistical analyses, confirm that the following items are present in the figure legend, table legend, main text, or Methods section.

n/a	Confirmed	
☐	☒	The exact sample size (*n*) for each experimental group/condition, given as a discrete number and unit of measurement
☐	☒	A statement on whether measurements were taken from distinct samples or whether the same sample was measured repeatedly
☐	☒	The statistical test(s) used AND whether they are one- or two-sided<br>*Only common tests should be described solely by name; describe more complex techniques in the Methods section.*
☐	☒	A description of all covariates tested
☐	☒	A description of any assumptions or corrections, such as tests of normality and adjustment for multiple comparisons
☐	☒	A full description of the statistical parameters including central tendency (e.g. means) or other basic estimates (e.g. regression coefficient) AND variation (e.g. standard deviation) or associated estimates of uncertainty (e.g. confidence intervals)
☐	☒	For null hypothesis testing, the test statistic (e.g. *F*, *t*, *r*) with confidence intervals, effect sizes, degrees of freedom and *P* value noted<br>*Give P values as exact values whenever suitable.*
☐	☒	For Bayesian analysis, information on the choice of priors and Markov chain Monte Carlo settings
☒	☐	For hierarchical and complex designs, identification of the appropriate level for tests and full reporting of outcomes
☐	☒	Estimates of effect sizes (e.g. Cohen's *d*, Pearson's *r*), indicating how they were calculated

Our web collection on statistics for biologists contains articles on many of the points above.

Software and code

Policy information about availability of computer code

Data collection	Neuralynx acquisition system (Cheetah 5.0); Blackrock Neurotech acquisition system; MATLAB Psychtoolbox version 3
Data analysis	'wave clus' MATLAB toolbox (version 3); Neural Decoding Toolbox (version 1.0.4); MATLAB 2019b; custom code using standard MATLAB functions (details in the text); MRIcroGL (version 1.2.20190902++Cocoa); BrainLab software (version 3.3.1.404), FSL (version 4.1), SPM (version 12)

For manuscripts utilizing custom algorithms or software that are central to the research but not yet described in published literature, software must be made available to editors and reviewers. We strongly encourage code deposition in a community repository (e.g. GitHub). See the Nature Portfolio guidelines for submitting code & software for further information.

Data

Policy information about availability of data

All manuscripts must include a data availability statement. This statement should provide the following information, where applicable:

- Accession codes, unique identifiers, or web links for publicly available datasets
- A description of any restrictions on data availability
- For clinical datasets or third party data, please ensure that the statement adheres to our policy

For consent and ethical consideration, the data supporting the findings of this study cannot be made publicly available but will be provided upon reasonable request to collaborative researchers.

Research involving human participants, their data, or biological material

Policy information about studies with human participants or human data. See also policy information about sex, gender (identity/presentation), and sexual orientation and race, ethnicity and racism.

Reporting on sex and gender	Throughout the text, we use the term 'sex' to refer to the participant's biological attribute (self-reported). We did not assess gender identity. We believe that the findings apply across the sexes similarly, thus, we have not included the participant's sex as a factor in any of the analyses (also because of the sample size). Sex and gender were not considered in the study design (i. e., recruitment was based solely on clinical criteria).
Reporting on race, ethnicity, or other socially relevant groupings	We have not grouped participants based on race, ethnicity, or other socially relevant criteria.
Population characteristics	The participants were 17 patients with intractable epilepsy who were implanted with depth electrodes to delineate a potentially surgically-treatable epileptogenetic zone (age: 21-69 y. o.; 10 females; Extended Data Table 1). Follow-up studies included 33 healthy controls (26 females; mean age: 31±7 years old) and 5 additional participants with epilepsy (2 females; mean age: 38±12 years old).
Recruitment	All patients undergoing the clinical monitoring procedure during the data collection period were invited to participate in the study. Due to the invasive nature of the intracranial recordings, only participants with intractable epilepsy were recruited. This could present some bias in the data.
Ethics oversight	UCLA Medical Institutional Review Board (IRB)

Note that full information on the approval of the study protocol must also be provided in the manuscript.

Field-specific reporting

Please select the one below that is the best fit for your research. If you are not sure, read the appropriate sections before making your selection.

☒ Life sciences ☐ Behavioural & social sciences ☐ Ecological, evolutionary & environmental sciences

For a reference copy of the document with all sections, see nature.com/documents/nr-reporting-summary-flat.pdf

Life sciences study design

All studies must disclose on these points even when the disclosure is negative.

Sample size	No statistical methods were used to predetermine sample size, as there are no previous studies that report similar effects. However, human single-neuron studies focusing on other mental processes have generally used similar sample sizes (e.g., Cerf et al. 2010, Nature; Kaminski et al., 2018, Current Biology; Bausch et al., 2021; Nature Communications, etc.).
Data exclusions	Data from all subjects and all recording sessions were included in the analyses.
Replication	The same experimental procedure was applied to all 17 participants in the main study (21 recording sessions). Group-level analyses suggest that the results are consistent across the subjects and replicable. The additional study conducted on additional 5 participants (7 recording sessions) used a slightly different procedure than the main study (i.e., different temporal structure) but the results were highly consistent, which provides an internal replication and generalization of our findings.
Randomization	There is only one experimental group in the study.
Blinding	n/a

Reporting for specific materials, systems and methods

We require information from authors about some types of materials, experimental systems and methods used in many studies. Here, indicate whether each material, system or method listed is relevant to your study. If you are not sure if a list item applies to your research, read the appropriate section before selecting a response.

Materials & experimental systems

n/a	Involved in the study
[x]	[ ] Antibodies
[x]	[ ] Eukaryotic cell lines
[x]	[ ] Palaeontology and archaeology
[x]	[ ] Animals and other organisms
[x]	[ ] Clinical data
[x]	[ ] Dual use research of concern
[x]	[ ] Plants

Methods

n/a	Involved in the study
[x]	[ ] ChIP-seq
[x]	[ ] Flow cytometry
[ ]	[x] MRI-based neuroimaging

Magnetic resonance imaging

Experimental design

Design type	Structural MRI and CT were used to localize the location of electrodes.
Design specifications	1 MRI and 1 CT image per subject.
Behavioral performance measures	We report reaction times and accuracy rates in the Extended Data Fig. 1. Median accuracy rates were high (above 80%), which shows that the subjects performed the tasks as expected.

Acquisition

Imaging type(s)	MRI (T1, T2) and CT
Field strength	3 Tesla
Sequence & imaging parameters	standard T1 and T2 weighted sequences
Area of acquisition	whole-brain

Diffusion MRI [ ] Used [x] Not used

Preprocessing

Preprocessing software	Each participant's MRI and CT images were co-registered using the FSL "flirt" function. The MRI image was then segmented into the grey matter, white matter, and cerebrospinal fluid probability maps, and resampled (1-1-1 mm voxel size).
Normalization	We used the 152 T1-weighted MNI template using the nonlinear transformation algorithm implemented in the Statistical Parametric Mapping toolbox (SPM12, Wellcome Department of Cognitive Neurology, London, UK).
Normalization template	see above
Noise and artifact removal	This was not necessary as the structural MRI was used only to localize the location of the electrodes.
Volume censoring	see above

Statistical modeling & inference

Model type and settings	No statistical modeling and inference was implemented, as the structural MRI was used only to localize the electrode locations.
Effect(s) tested	see above

Specify type of analysis: [x] Whole brain [ ] ROI-based [ ] Both

Statistic type for inference (See Eklund et al. 2016)	see above
Correction	see above

Models & analysis

n/a	Involved in the study
☒	☐ Functional and/or effective connectivity
☒	☐ Graph analysis
☒	☐ Multivariate modeling or predictive analysis
